# Supplementary material for: Variable efficiency of nonsense-mediated mRNA decay across human tissues, tumors and individuals
Source: Genome Biol. 2025 Sep 29;26:316. doi: 10.1186/s13059-025-03727-y (PMC12477815; doi:10.1186/s13059-025-03727-y)
Supplement: Supplementary file 1 — Additional File 1: Supplementary Figures S1-S29 [file 13059_2025_3727_MOESM1_ESM.pdf]

Supplementary Figures S1-S29 for Palou and Supek (2025) *Genome Biology*.

Fig. S1

A

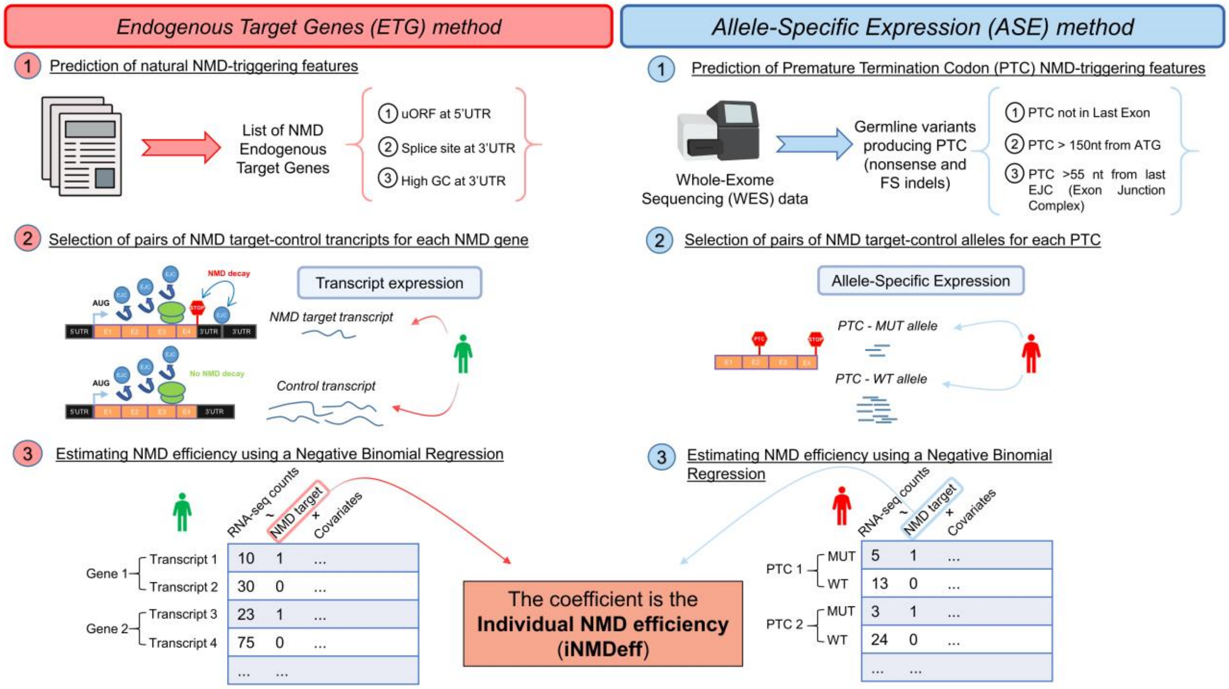

B

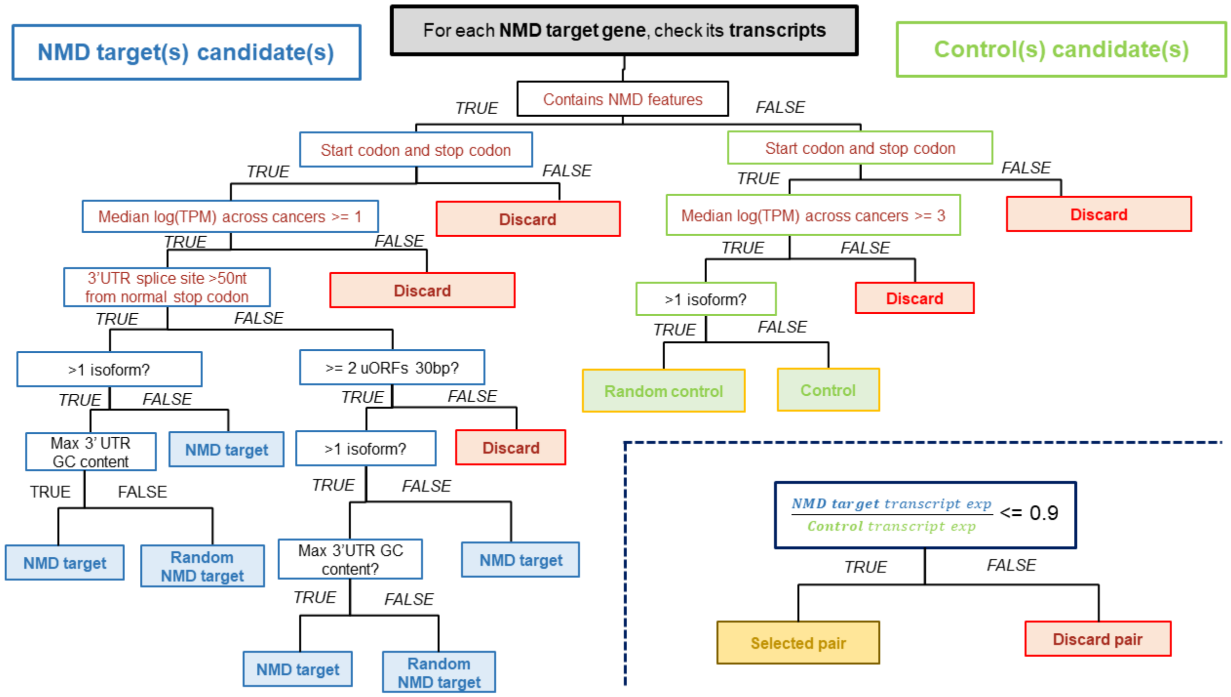

Fig S1. Overview of NMD methods to estimate individual NMD efficiency (iNMDeff).

A, *Schematic illustration of the two orthogonal methodologies employed to estimate iNMDeff: the Endogenous Target Gene (ETG) NMD method, on the left, and the Allele-Specific Expression (ASE) NMD method, on the right. Each method provides a unique approach to quantify the effectiveness of the NMD pathway in degrading mRNA transcripts for each individual, either using germline premature termination codons (PTCs) or endogenous transcripts with NMD-triggering features (see Methods for details).* ***B****, Methodology for pairing NMD target and control transcripts for the ETG method. Decision tree outlining the process for selecting a pair of NMD target and control transcripts for each gene within the various NMD gene sets for the ETG method. The left branch of the tree delineates criteria for identifying NMD target candidates based on intrinsic NMD-triggering features: i) a splice site in the 3' untranslated region (UTR) situated at least 50 nt downstream from the normal stop codon; ii) an upstream open reading frame (uORF) within the 5' UTR; iii) high GC content within the 3'UTR. If multiple transcripts per gene exhibit these features, one is chosen at random. The right branch of the tree defines the selection of control candidates, which must exhibit some level of gene expression across cancer types and being absent of NMD-triggering features. After pairing an NMD target with a control, the transcript expression ratio in wild-type cell lines must be less than 0.9 to be valid. If this criterion is not met, the selection process is reiterated to identify an alternative pair.*

Fig. S2

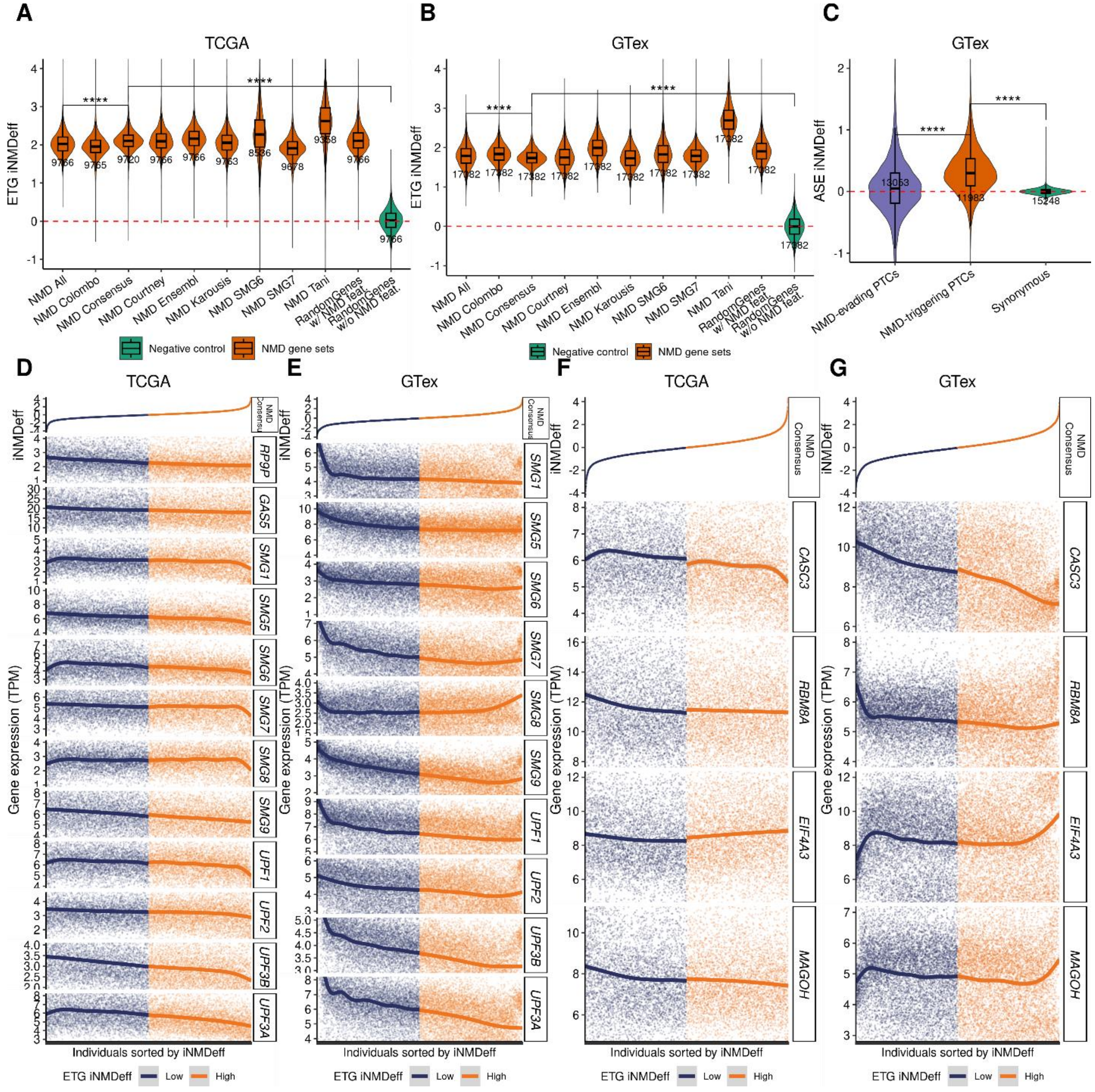

Fig. S2. Individual-level quantification of NMD efficiency.

A-B, *Estimation of individual NMD efficiency -- iNMDeff -- using the ETG method across 9,766 TCGA samples (A) and 17,382 GTex samples (B), showcasing all NMD gene sets, one random gene set with NMD-triggering features (RandomGenes w/ NMD feat.), and one random gene set without NMD-triggering features as negative control (RandomGenes w/o NMD feat.).* ***C***, *iNMDeff estimations using the ASE method, in GTex, for two NMD variant sets: NMD-evading and NMD-triggering PTCs, alongside a non-NMD variant set as a negative control (Synonymous). ****$p < 0.0001$, by two-sided Mann–Whitney U test for A-C.* ***D-E,*** *Gene expression levels (TPM) of 9 core NMD factors (SMG1, SMG5, SMG6, SMG7, SMG8, SMG9, UPF1, UPF3A, UPF3B) and two well-known NMD targets (RP9P and GAS5, here for TCGA only) compared against the ETG iNMDeff (using the "NMD Consensus" gene set) sorted from lowest to highest along the X-axis in TCGA (D) and GTex (E). Samples are stratified by the median ETG iNMDeff in high and*

low. ***F-G****, Same as in D-E but for 4 core exon-junction complex (EJC) genes (CASC3, RBM8A, EIF4A3, MAGOH) in TCGA (F) and GTex (G). TCGA: The Cancer Genome Atlas, GTex: Genotype-Tissue expression, PTC: Premature Termination Codon. NMD: nonsense-mediated mRNA decay.*

Fig. S3

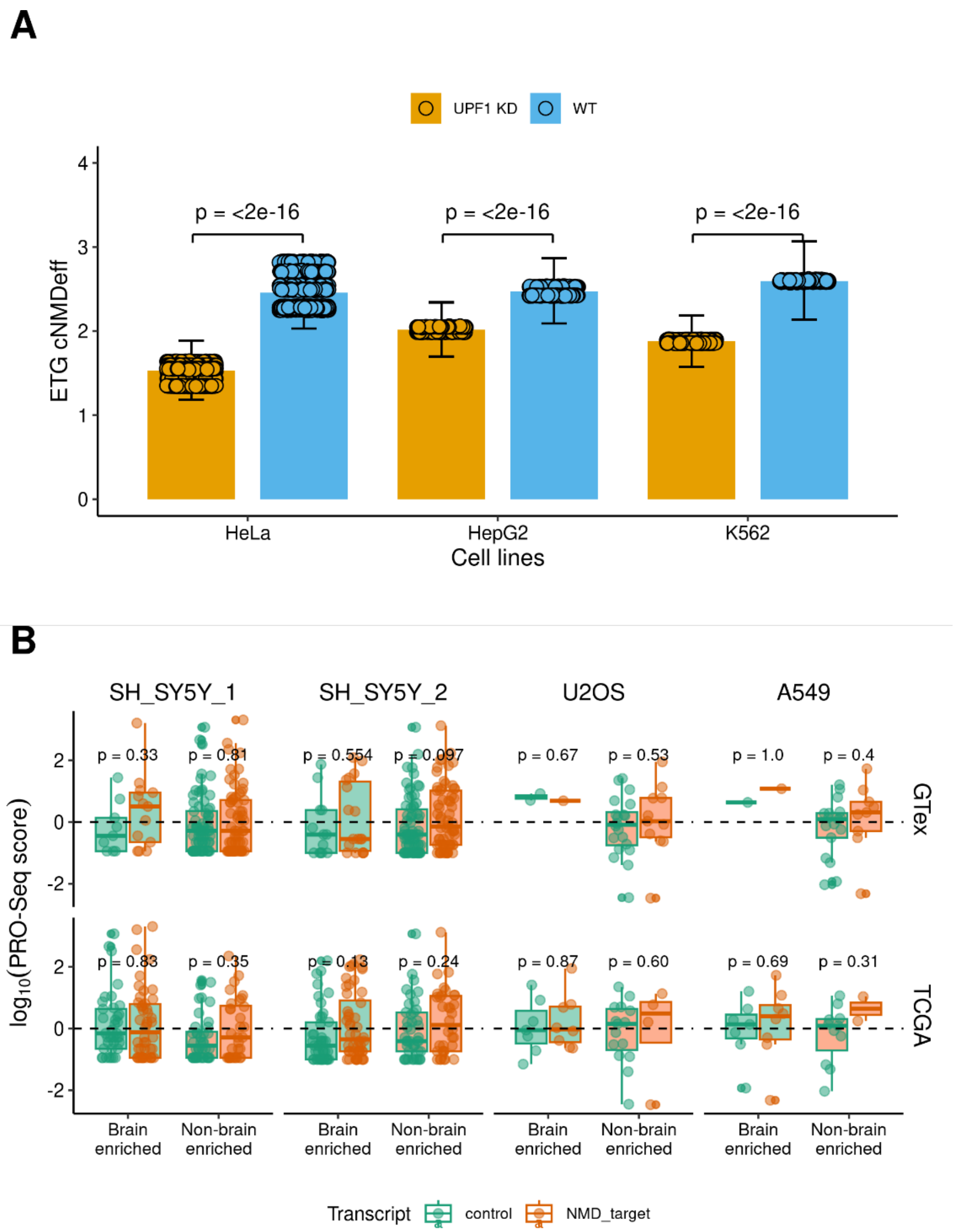

Fig. S3. Validation of the ETG iNMDeff method.

A, *Cell line ETG NMD efficiency (cNMDeff) in HeLa (n = 8), HepG2 (n = 2), and K562 (n = 2) cell lines using the "NMD Consensus" gene set. cNMDeff was calculated using negative binomial regression, similar to our iNMDeff analysis (see Methods). A leave-one-out validation was performed on 130 NMD Consensus genes, with sequential removal of one gene at a time followed by cNMDeff recalculation. Comparison between UPF1 knockdown (KD) and wild-type (WT) conditions showed consistent reduction in NMD efficiency across all cell lines, independent of gene outliers. Barplots and 95% confidence intervals represent the mean across cells.* ***B***, *Transcriptional output analysis using PRO-seq scores in A549 (n = 1), U2OS (n = 1), and SH-SY5Y (n = 2 experiments; SH-SY5Y_1 and SH-*

SY5Y_2) cell lines. Promoter activity was compared between NMD-target and non-target (control) transcripts from the ETG Consensus gene set, measuring PRO-seq signals at TSS and downstream regions (see Methods). Brain-specific gene expression enrichment was assessed by comparing mean gene TPM values between merged normal brain subregions and all other tissues (GTex, top row) or between LGG/GBM tumors and other cancers (TCGA) (bottom row) to define brain-enriched genes (performing Mann-Whitney U test, alternative = greater, $p < 0.05$). P-values on plot are calculated by Mann-Whitney U test.

Fig. S4

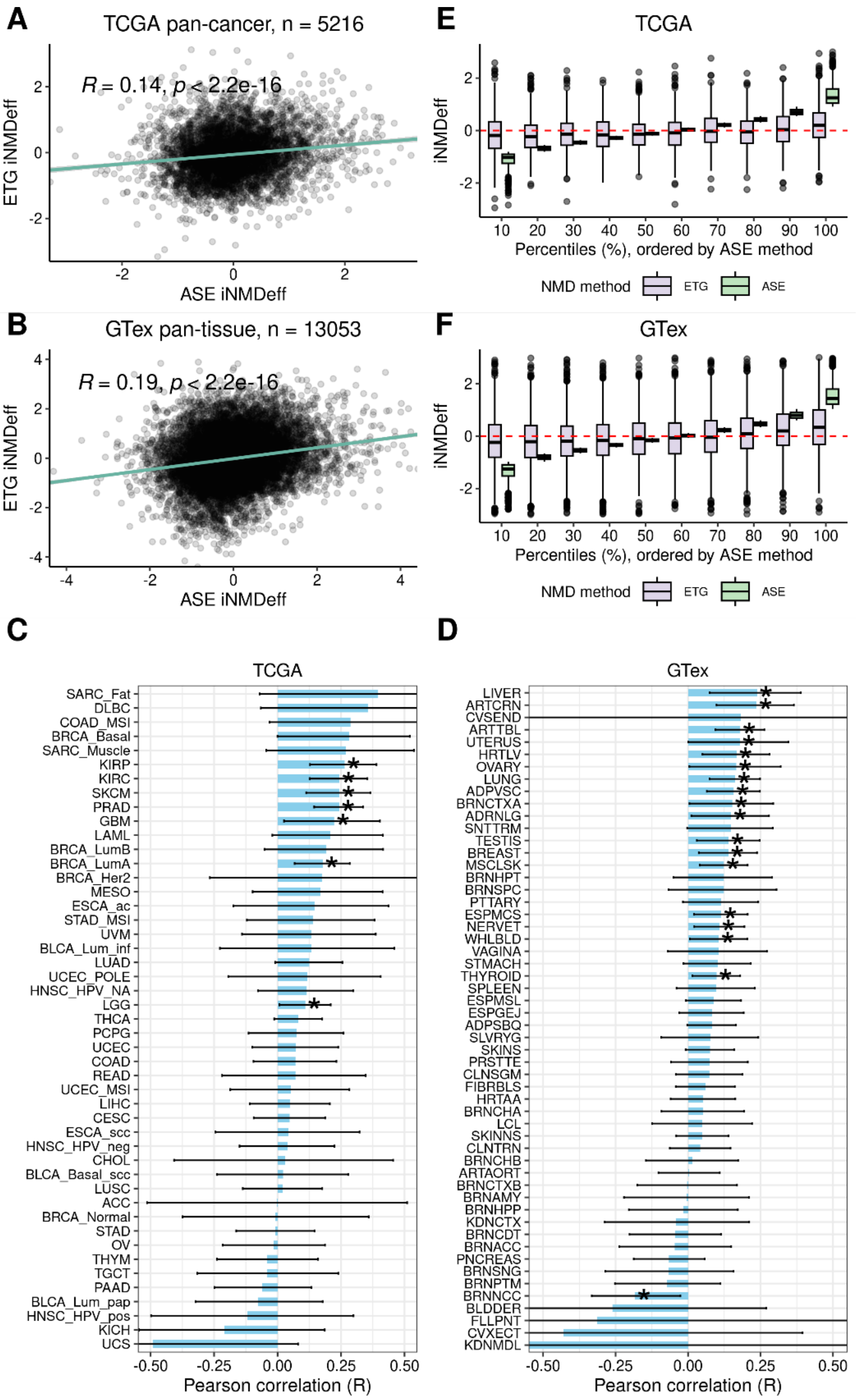

Fig. S4. Agreement between the two methods to estimate iNMDeff.

A-B, *Correlation between ETG iNMDeff and ASE iNMDeff across pan-cancer samples in TCGA (A) and pan-tissue samples in GTex (B). Due to missing ASE data because of the stringent filterings (see Methods), the number of samples analyzed in TCGA is 5,216 and 13,053 in GTex. Each plot displays*

the Pearson correlation coefficient (R) and associated p-value. ***C-D****, Similar to A-B, but stratified for each cancer type (or subtype) in TCGA (C) and each normal tissue in GTex (D). The X-axis shows the Pearson correlation coefficient (R), along with its 95% confidence interval, with the range limited to between -0.5 and 0.5 for clarity. Correlations deemed significant (FDR < 5%) are marked with an asterisk (*).* ***E-F****, Stratification of iNMDeff into ten deciles for both ASE and ETG methods, but ordered only by the ASE method, for TCGA (F) and GTex (G). It also demonstrates the concordance between the two approaches.*

Fig. S5

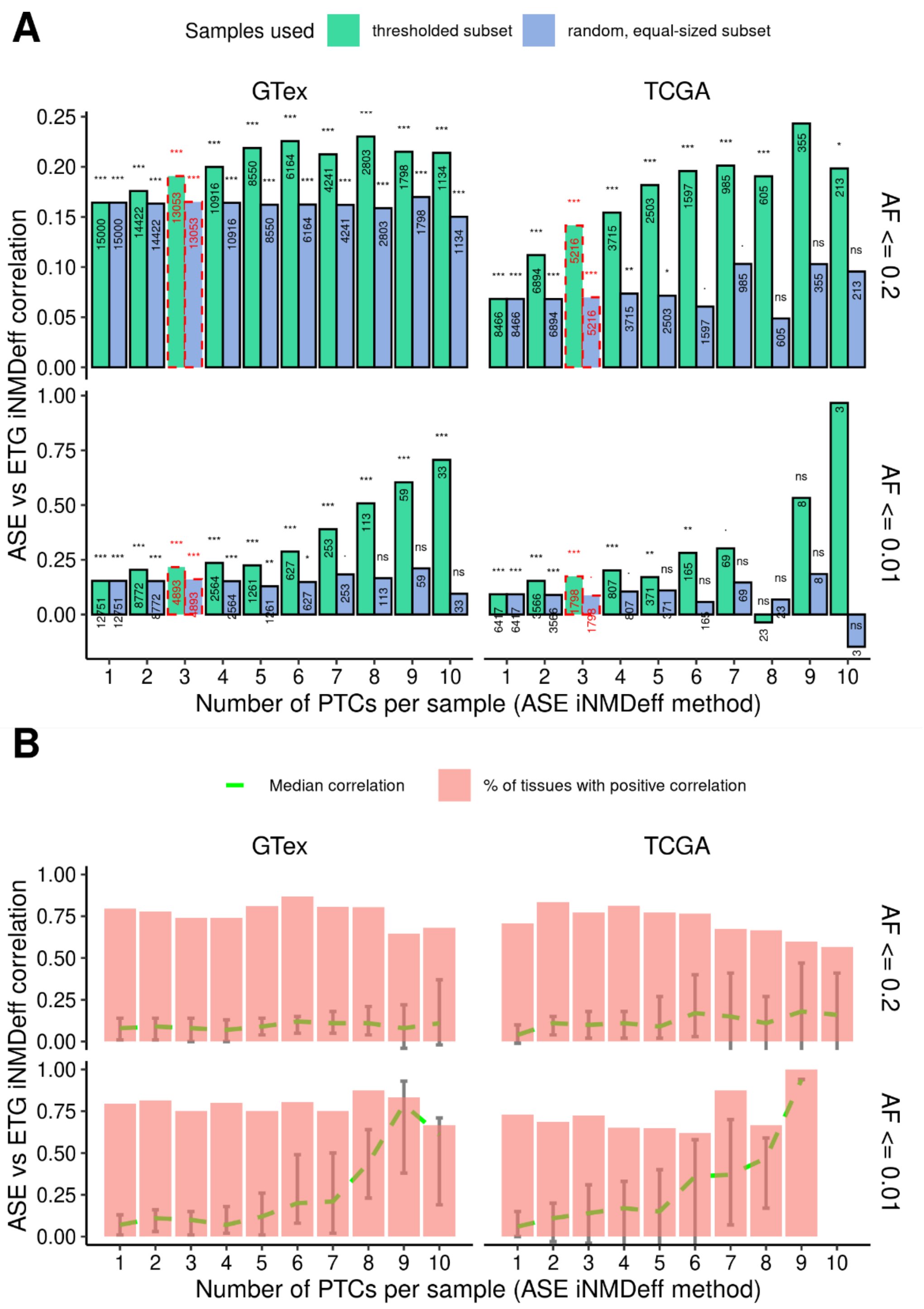

Fig. S5. Stringent ASE filterings enhance correlations with ETG iNMDeff.

A, *Pearson correlations (R) between ASE and ETG iNMDeff methods, at individual sample-level, across different filtering criteria in GTex (pan-tissue, left panel) and TCGA (pan-cancer, right panel). Two filtering parameters were varied: i) minimum number of germline PTC variants per sample*

*required for ASE iNMDeff estimation (X-axis), and ii) PTC population allele frequency (AF) threshold (AF ≤0.2 in upper panels; AF ≤0.01 for strictly rare variants in bottom panels). Numbers above bars indicate the total number of samples remaining after applying filters. Under current thresholds (≥3 PTCs per sample and AF ≤0.2), 13,053 (75%) of GTex samples and 5,216 (53%) of TCGA samples are retained. Statistical significance: *** p < 0.001, ** p < 0.01, * p < 0.05, ns = non-significant.* ***B***, *Tissue-specific analysis using the same sample filtering criteria as in (A), showing correlations calculated separately for each normal tissue (GTex) or cancer type (TCGA). We show the median correlation with Q1-Q3 range as whiskers, and the proportion of tissues/cancers displaying positive correlation as barplots. Both results (A and B) demonstrate a trade-off between precision and sample retention.*

Fig. S6

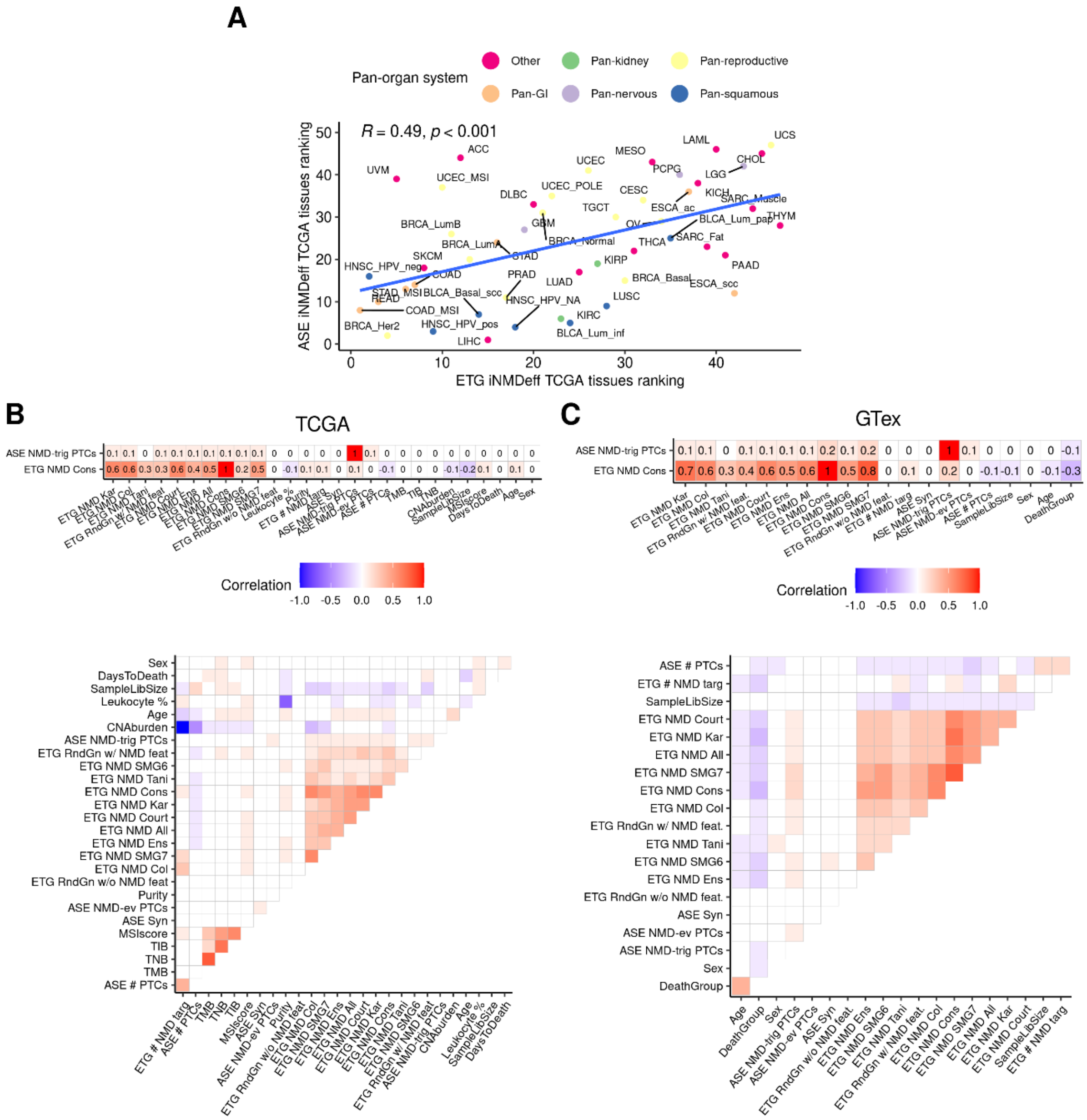

Fig. S6. Correlations between ASE and ETG iNMDeff including technical and biological covariates.

A, *Spearman correlation between cancer type rankings based on median ETG iNMDeff and median ASE iNMDeff values, for the TCGA cohort. Cancers are grouped based on cell-of-origin: Nervous system-related tissues (Pan-nervous), Kidney-related tissues (Pan-kidney), Reproductive system tissues (Pan-reproductive), Gastrointestinal tissues (Pan-GI), and those originating from Squamous cells (Pan-squamous).* ***B-C****, Correlations between various biological and technical variables and iNMDeff estimates from both ASE and ETG methods for TCGA (A) and GTex (B) cohorts. For clarity, the top panels includes focused correlations, highlighting the "NMD Consensus" (ETG method) and "NMD-triggering PTCs" (ASE method) correlations with all variables, while the bottom panels show the complete correlation matrices with all pairwise variable relationships. Biological variables include: sex, leukocyte %, days to death (TCGA), death group (Gtex), age, somatic copy number alteration*

burden (CNAburden, TCGA), microsatellite-instability score (MSIscore, TCGA), tumor indel burden (TIB, TCGA), tumor mutation burden (TMB, TCGA), tumor nonsense burden (TNB), number of PTCs used for the ASE method (ASE # PTCs) and number of NMD targets used for the ETG method (ETG # NMD targ). Technical variables include: RNA-seq sample library size (SampleLibSize) and tumor purity (TCGA). ETG iNMDeff estimates from 11 NMD gene sets, including its 2 non-NMD controls, are: ETG NMD SMG6, ETG NMD SMG7, ETG NMD Tani, ETG NMD Consensus, ETG NMD Karousis, ETG NMD Courtney, ETG NMD All, ETG NMD Ensembl, ETG NMD Colombo, ETG NMD RandomGenes without NMD features (ETG NMD RndGn w/o NMD feat), ETG NMD RandomGenes with NMD features (ETG NMD RndGn w/ NMD feat). ASE iNMDeff estimates from 3 NMD variant sets, including its controls are: ASE NMD-triggering PTCs, ASE NMD-evading PTCs and ASE Synonymous. The Pearson correlation coefficient (R) is represented by a color spectrum ranging from -1 (blue) for a perfect negative correlation to 1 (red) for a perfect positive correlation with iNMDeff.

Fig. S7

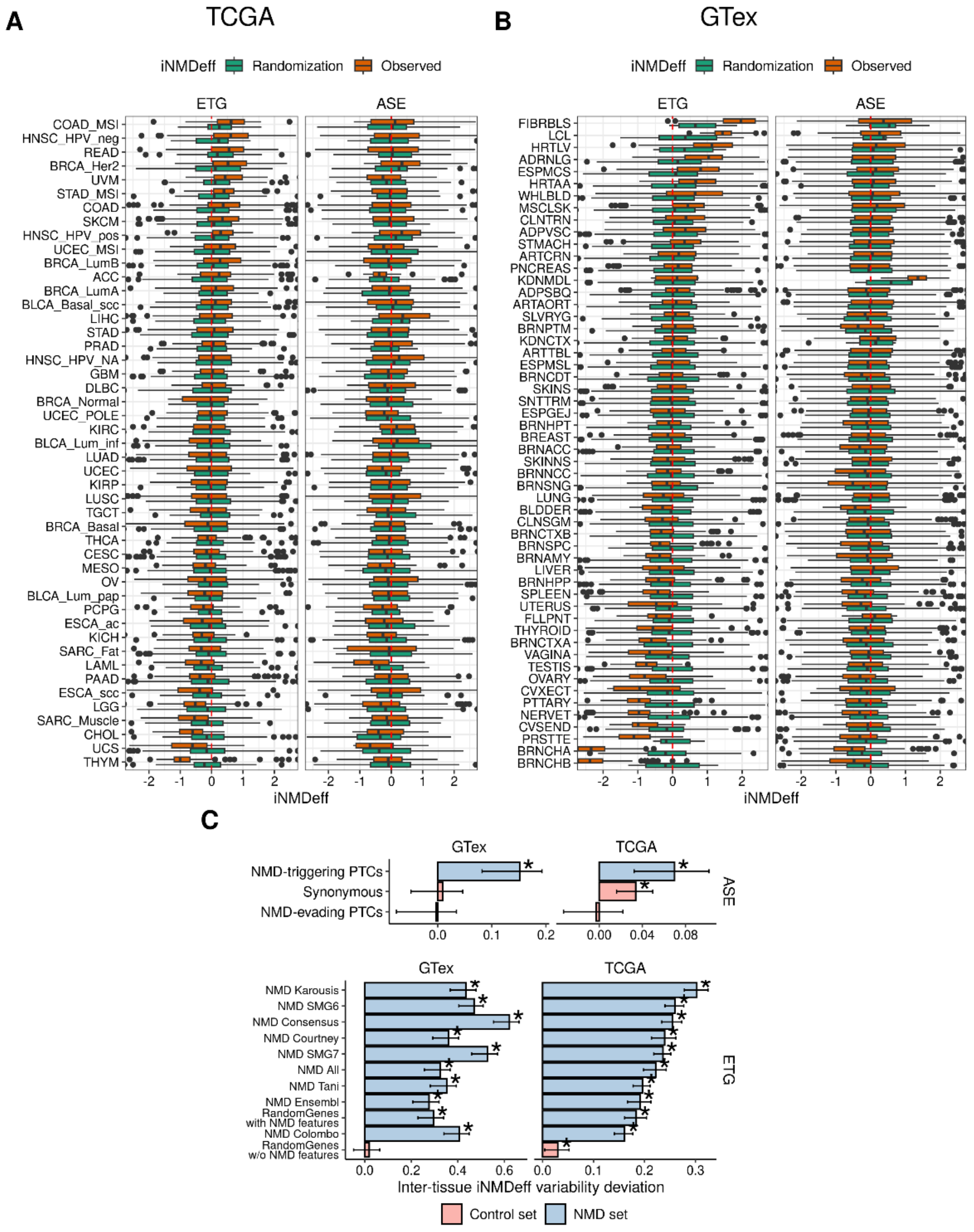

Fig. S7. Tissue iNMDeff values and inter-tissue variability deviation of NMD efficiency. ***A-B***, *Distribution of iNMDeff values for both ETG (left panel) and ASE (right panel) methods across cancer types (or subtypes) in TCGA (A) and normal tissues in GTex (B) are displayed on the X-axis as orange boxplots. Corresponding randomized iNMDeff values are illustrated alongside as green boxplots. Complete names of tissue acronyms can be found in Additional File 2: Table S5.* ***C***, *The Inter-Tissue iNMDeff Variability Deviation (ITNVD) test scores are shown for all evaluated NMD gene sets (11 for ETG method, 3 for ASE method), indicating the extent of variability in iNMDeff. For contrast, scores for non-NMD control gene/variant sets are also presented. Results from both GTex (left) and TCGA (right) cohorts are included. Positive ITNVD scores suggest significant variability in*

iNMDeff among different tissues or cancers, with statistical significance ($p \leq 0.05$) denoted by stars (*), as determined by the randomization test.

Fig. S8

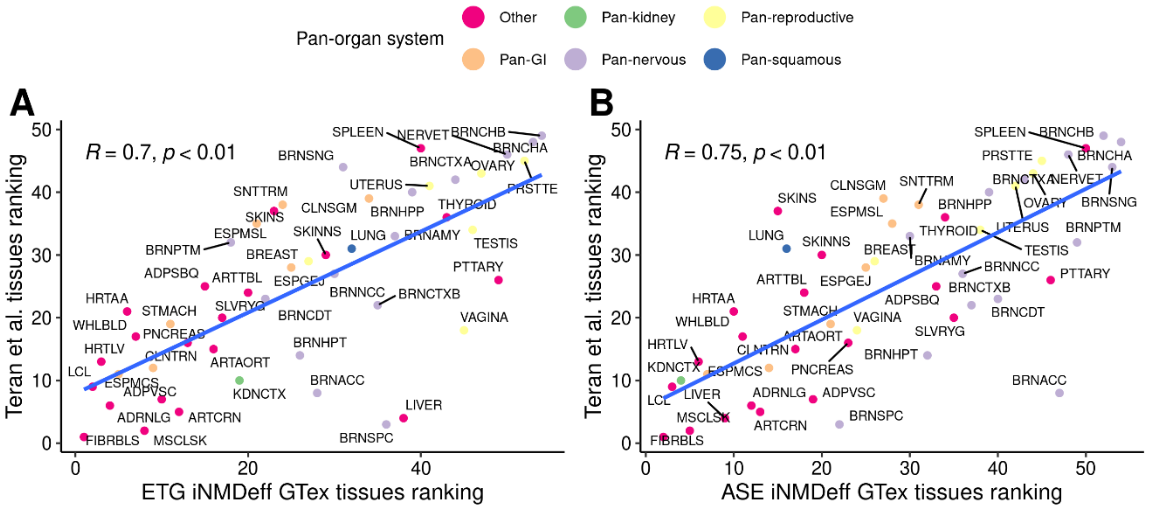

Fig. S8. Tissue-specific NMD efficiency rankings.

A-B*, Correlations of the ranking of tissues based on the median ETG iNMDeff (A) and ASE iNMDeff (B) from this study with the tissue ranking derived from Teran et. al. 2021. using ASE PTCs-NMDeff methodology. The analysis provides Spearman correlations and corresponding p-values, quantifying the agreement between the two independent tissue ranking approaches. Tissues are grouped based on cell-of-origin: Nervous system-related tissues (Pan-nervous), Kidney-related tissues (Pan-kidney), Reproductive system tissues (Pan-reproductive), Gastrointestinal tissues (Pan-GI), and those originating from Squamous cells (Pan-squamous) for panels A-B.*

Fig. S9

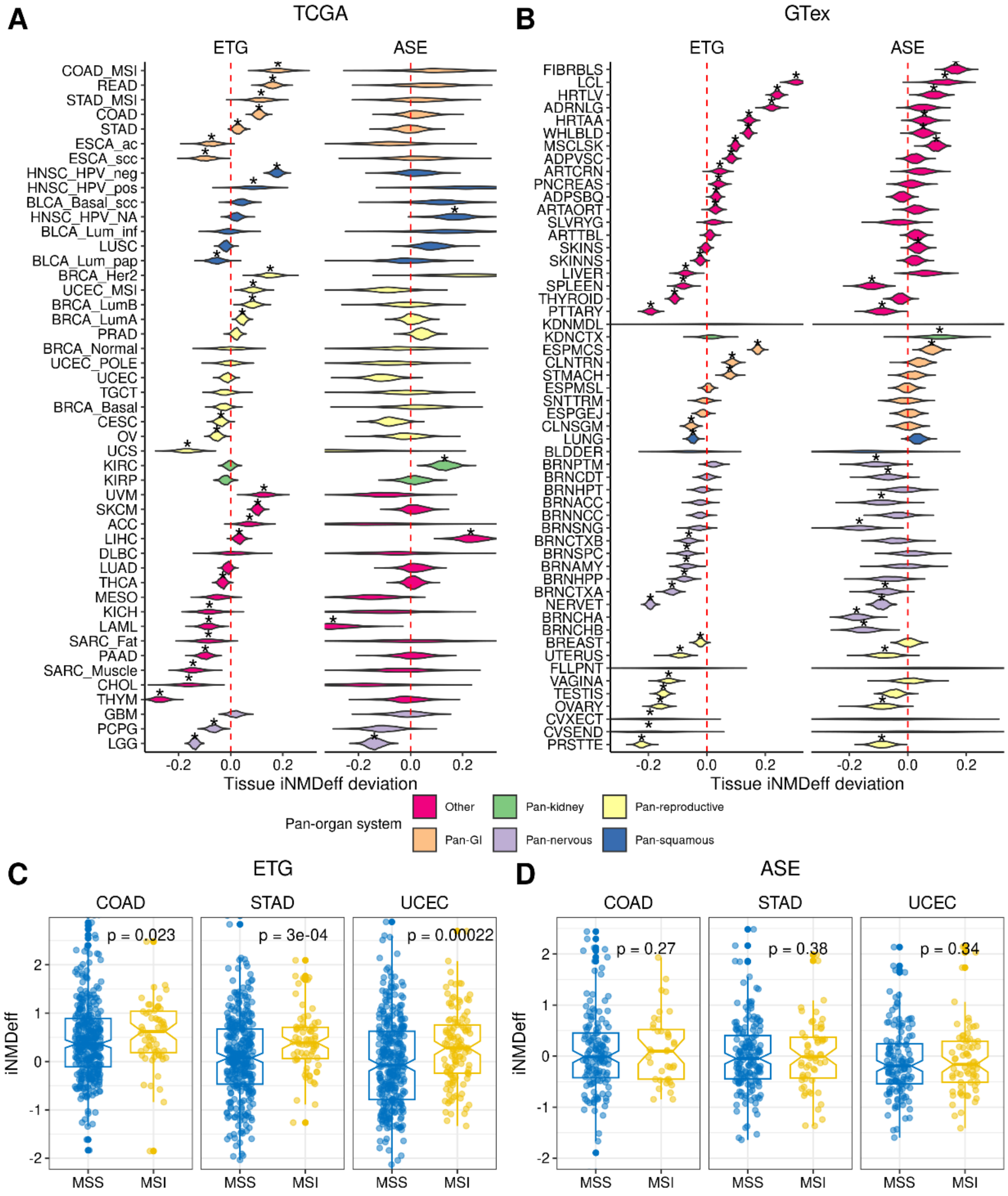

Fig. S9. Significant differences between tissues in NMD efficiency.

A-B, *Shows Tissue iNMDeff Deviation (TND) test scores across various cancer types in TCGA (A) and normal tissues in GTex (B), providing insight into the specific variations of NMD efficiency within tissues. Tissues are classified in primary groups: Nervous system-related tissues (Pan-nervous), Kidney-related tissues (Pan-kidney), Reproductive system tissues (Pan-reproductive), Gastrointestinal tissues (Pan-GI), those originating from Squamous cells (Pan-squamous), and the remaining tissues (Other). The groups of tissues were ordered based on the median TND scores, arranging them from the highest to the lowest median scores, top to bottom.* ***C-D***, *Displays the iNMDeff for both ETG (C) and ASE (D) methods in TCGA cancers: colon adenocarcinoma (COAD),*

uterine corpus endometrial carcinoma (UCEC), and stomach adenocarcinoma (STAD), further dividing samples into microsatellite instability (MSI) and microsatellite stable (MSS) groups. One-sided Mann-Whitney U tests were used to calculate p-values.

Fig. S10

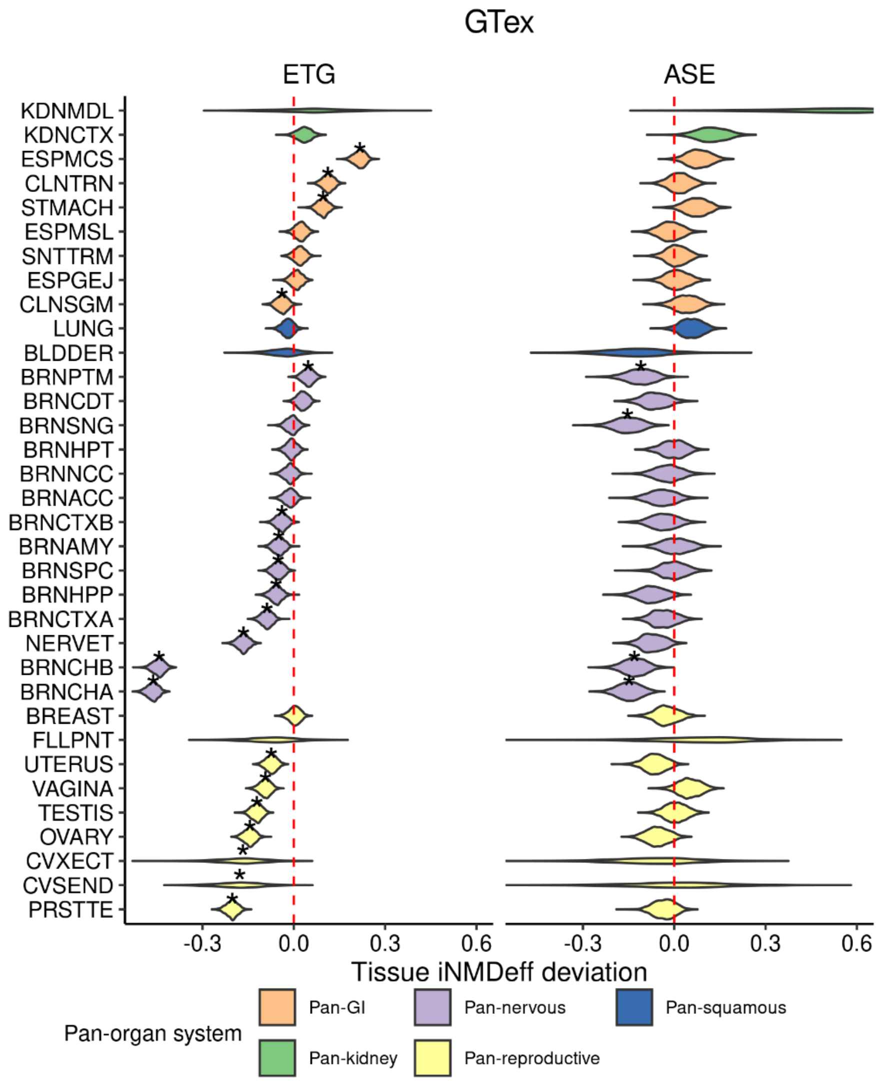

Fig. S10. Significant inter-tissue variability of NMD efficiency after subsampling in GTex.

Tissue iNMDeff Deviation (TND) analysis performed on subsampled tissues as in the original (Fig. S9), in order to control for sample size differences between brain subregions (n=139-255, mean=203) and the rest of tissues (n=4-802, mean=359). Results showed a consistent proportion of brain tissues with significant differences (FDR < 0.05) between subsampled (8/13 brain tissues) and original (7/13) analyses (Fig. S9). The subsampling was done per tissue as to match the sample size of the smallest brain subregion (BRNSNG, n=139), except for tissues already below this threshold (KDNMDL, CVXECT, FLLPNT, CVSEND, BLDDER, KDNCTX).

Fig. S11

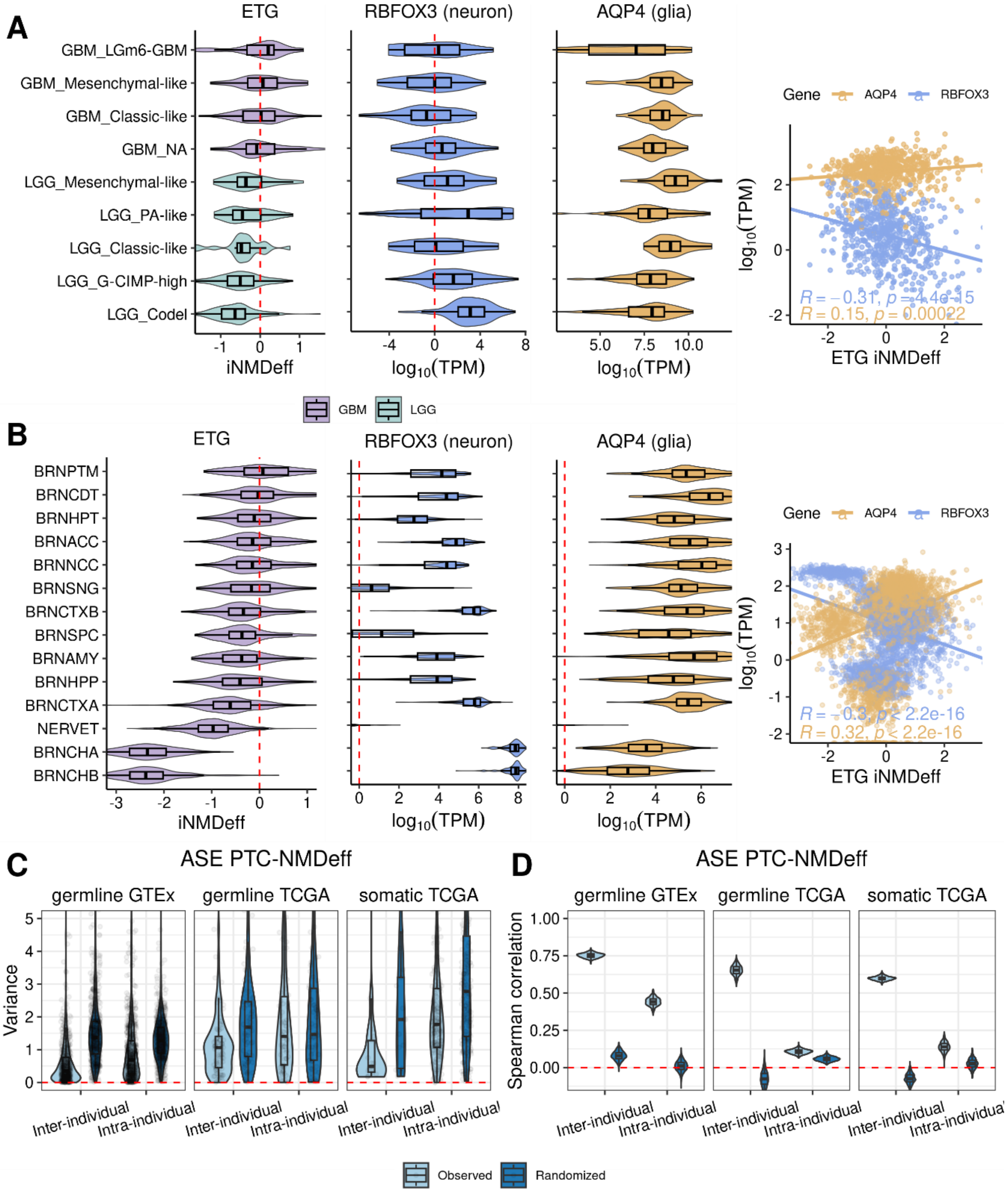

Fig. S11. Lower NMD efficiency in the tissues of the nervous system and extensive inter-individual variation of NMD efficiency.

A, *Scaled ETG iNMDeff for LGG and GBM within the TCGA cohort, segmented by genetic or histological subtypes (first panel). This section also shows the scaled gene expression (TPM) of the neural marker gene RBFOX3 (second panel), the glial gene marker AQP4 (third panel), and their Pearson correlations (R) with ETG iNMDeff (fourth panel).* ***B,*** *Mirrors the analysis in A but focuses on GTex brain tissues, stratified by different subregions, showcasing the variability of NMD efficiency in normal brain tissue contexts and its correlation with neuron or glial cell type gene expression markers.*

C-D*, Intra- and inter-individual variability of iNMDeff, leveraging ASE PTC-NMDeff. C assesses variability through the variance of all PTCs within individuals (intra-individual variability) and between all individuals sharing the same PTC (inter-individual variability). D, repeats this comparative analysis using Spearman correlation instead of variance to measure variability. For intra-individual variability we randomly selected pairs of PTCs, and for inter-individual variability we selected pairs of randomly chosen individuals sharing the same PTC. Light blue boxplots represent observed values, while dark blue boxplots serve as randomization controls, providing a baseline for comparison. These analyses are conducted within the TCGA cohort for both germline and somatic PTCs and extended to germline PTCs within the GTex cohort (see Methods).*

Fig. S12

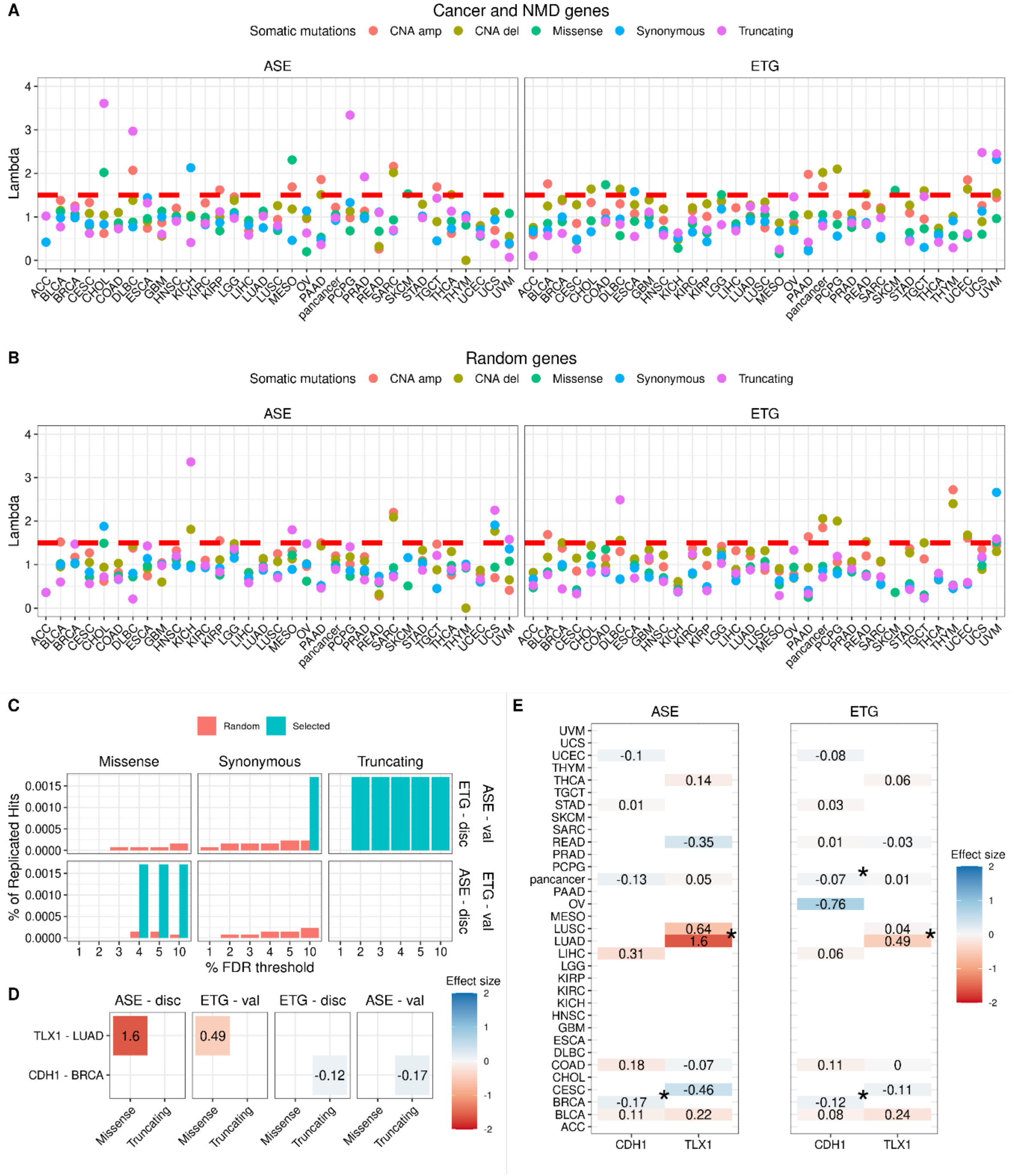

Fig. S12. Associations analysis of somatic mutations with NMD efficiency.
***A**, Shows inflation factor values (lambda) reflecting the degree of inflation in linear association studies between iNMDeff and various types of somatic mutations within cancer genes (TSGs and OGs) and NMD-related genes. Mutation types include Missense, Synonymous, Truncating, CNA amplifications, and CNA deletions. Analysis spans pan-cancer and individual cancer types for ASE (left panel) and ETG (right panel) methods. A horizontal dashed-red line marks our lambda threshold (>= 1.5), beyond which associations are considered excessively inflated and thus excluded from further analysis. **B**,*

Replicates the lambda figure from A but focuses exclusively on the remaining genes, effectively filtering out cancer and NMD-related genes to assess inflation in a control random gene set. ***C****, Proportion of replicated hits—calculated as the number of hits over the total number of tests—for each mutation type, across different FDR thresholds from 1% to 10%. The analysis alternates between ASE and ETG methods for discovery and validation within the same cancer type, illustrated in two panels: ETG discovery to ASE validation (top) and vice versa (bottom). Blue bars represent observed hits in cancer/NMD-related genes; red bars correspond to hits among the random genes. CNA amplifications/deletions were ultimately excluded due to significant inflation.* ***D****, Lists replicated and significant genes, categorized by somatic mutation type, and stratified by NMD method (ASE or ETG) and direction of discovery/validation. Effect sizes, shown as beta coefficients from the linear model associations with iNMDeff, are color-graded: negative values in red and positive in blue.* ***E****, Effect sizes of associations for all cancer types in TCGA for the two significant and replicated genes, using the same color gradient as in D for effect size visualization. Cancer-type specific associations marked with significance ($p < 0.05$) are highlighted with an asterisk (*).*

Fig. S13

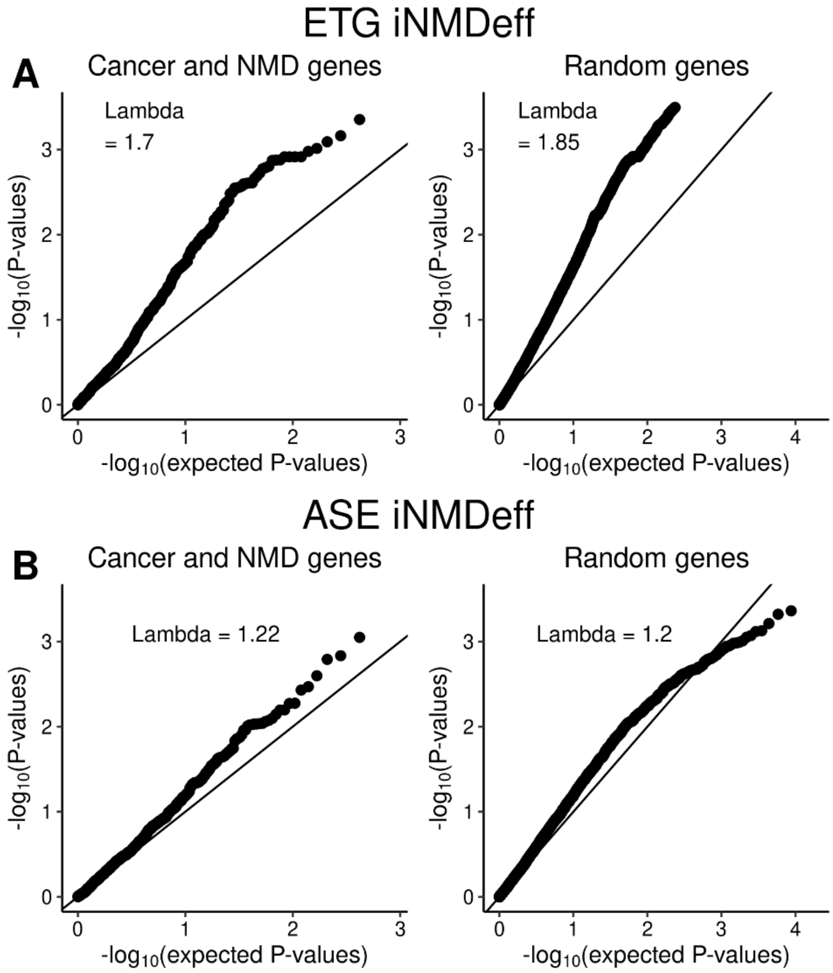

Fig. S13. Inflation factor of the associations between somatic gene-level CNA amplifications and iNMDeff.

A-B, *QQ plots examining the inflation in linear association studies between somatic gene-level CNA amplifications and iNMDeff, utilizing both ETG (A) and ASE (B) methods at the pan-cancer level. The analysis divides into associations within cancer and NMD-related genes (left panel) versus the remaining genes (right panel), with the Y-axis depicting the observed p-values and the X-axis the expected p-values, both in -log_{10} scale, to highlight deviation from expectation. Lambda values, indicate systemic bias, particularly in the ETG method, suggesting potential confounding effects in these associations.*

Fig. S14

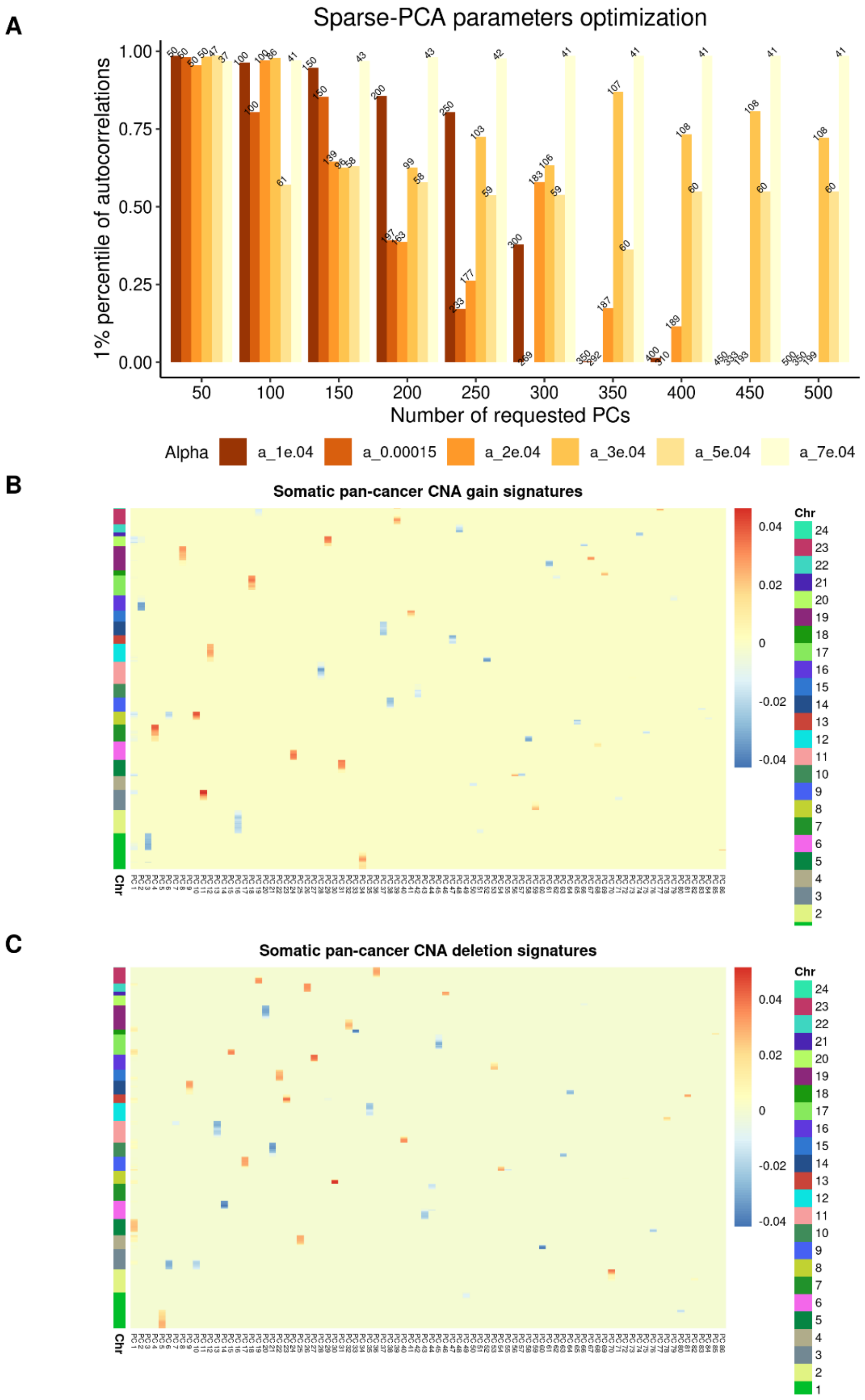

Fig. S14. Refinement of sparse principal component analysis (sparse-PCA) parameters to effectively capture arm-level CNAs.

***A**, Illustrates the number of principal components (PCs) derived from sparse-PCA, capturing CNA gains or losses, against autocorrelation scores for the 1% of PCs with the lowest autocorrelation. The Y-axis tracks autocorrelation values from 0 to 1 for these PCs, while the X-axis varies the number of requested PCs in the sparsepca function, ranging from 50 to 500. Different alpha parameters (from 7e-04 in red to 1e-04 in light yellow) dictate the color gradient, indicating the degree of sparsity. Atop each bar, the count of "effective" PCs—those not entirely composed of zeros in gene weights—is noted. Optimal parameters were identified as requesting 100 PCs, yielding 86 non-zero effective PCs, with an average autocorrelation of 0.99 among the top 1% of PCs. **B-C**, Heatmaps displaying the gene weights across all 86 pan-cancer CNA-PCs, from -0.04 (blue) to 0.04 (red), sorted by genomic and chromosomal location, with each chromosome distinctly colored (legend provided). Chromosome X is marked as 23, and Chromosome Y as 24. In B (for CNA gains) and C (for CNA deletions), the initial 47 CNA-PCs predominantly indicate broad-scale alterations, akin to arm-level changes, while the remaining PCs highlight more localized genomic events.*

Fig. S15

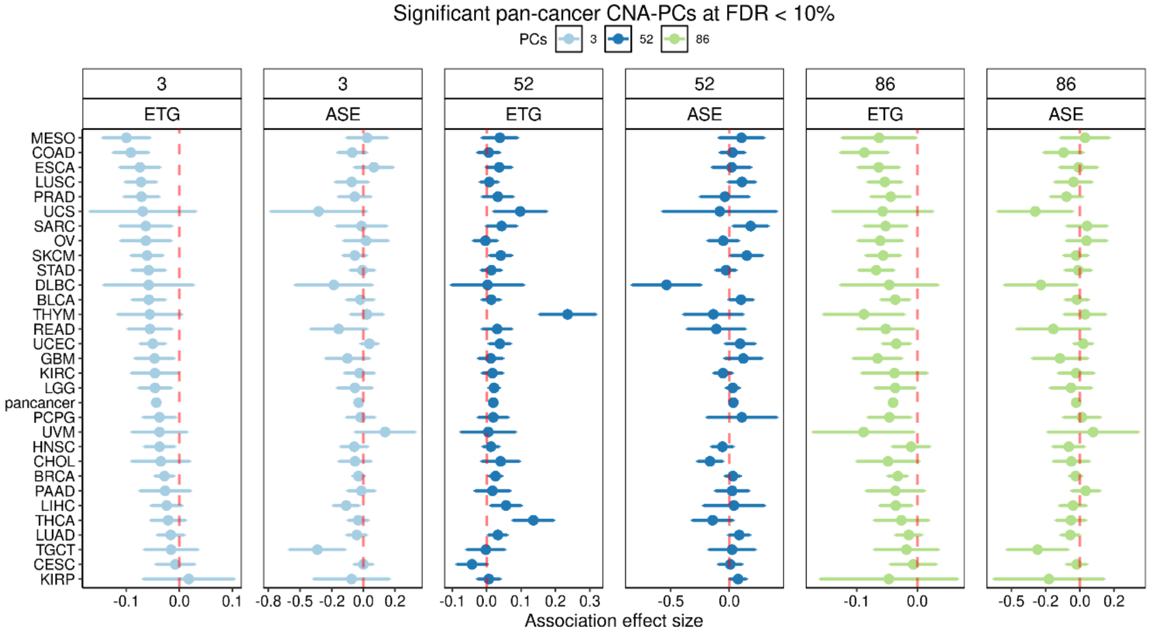

Fig. S15. Associations between replicated pan-cancer CNA-PCs and iNMDeff in TCGA.

Cancer-type and pan-cancer associations between three replicated pan-cancer CNA-PCs and iNMDeff, employing both ETG (left panels) and ASE (right panels) methodologies, under a 10% FDR threshold. The implicated CNA-PCs are CNA-PC 3, CNA-PC 52, and CNA-PC 86, each demonstrating a significant link with iNMDeff. The effect size of the associations (beta coefficient from the linear model) means that higher values of a given CNA-PC signature correlate with increased iNMDeff, and vice versa.

Fig. S16

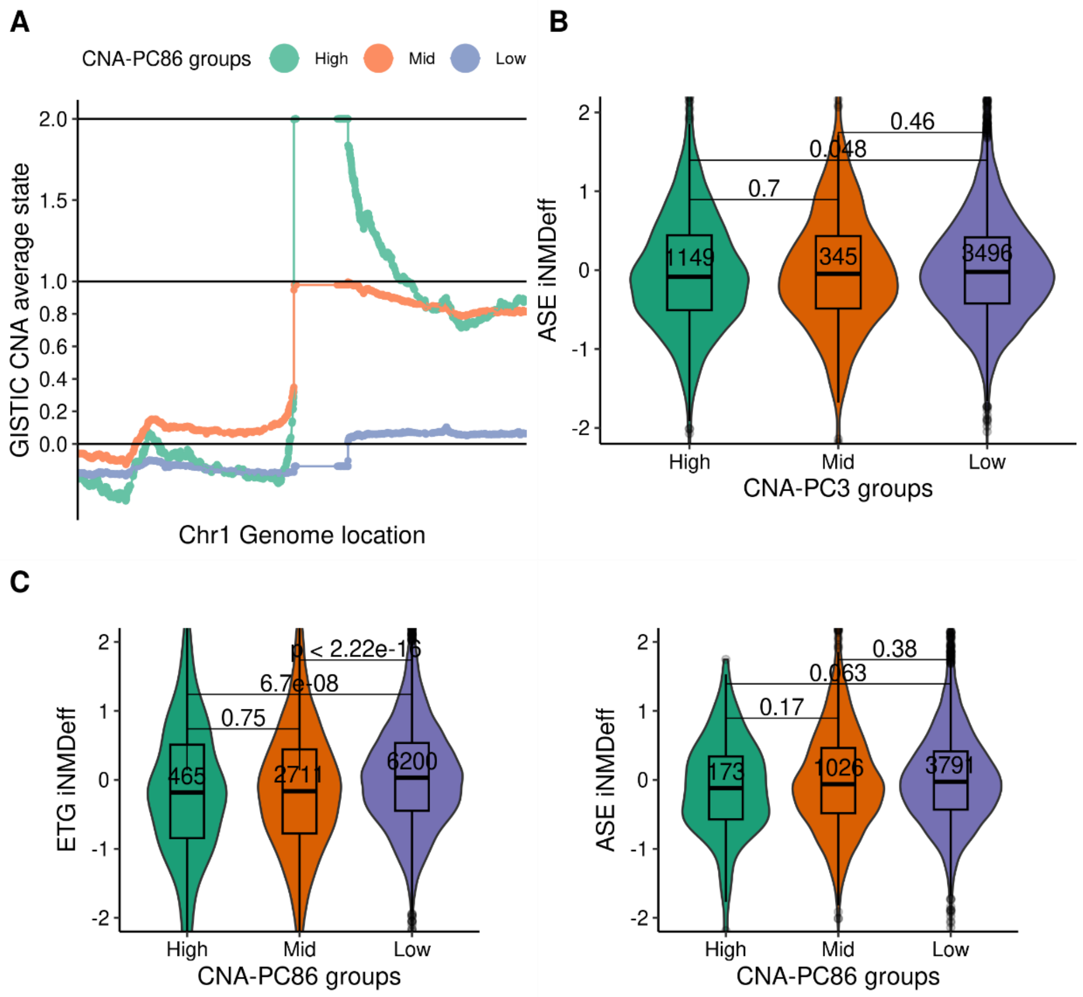

Fig. S16. Somatic chromosome 1q gains associate with reduced NMD efficiency.
A, *Gene-level amplifications across chromosome 1, ordered by genome location, assessed by averaging GISTIC CNA scores for each gene across TCGA participants. Samples are classified into three group bins based on their pan-cancer CNA-PC3 scores: "High", "Mid", and "Low".* ***B****, Stratification of scaled ASE iNMDeff according to these CNA-PC3 groupings: "High", "Mid", and "Low".* ***C****, Same as B but for ETG (left panel) and ASE (right panel) iNMDeff for samples grouped by CNA-PC86 scores bins. Statistical comparisons between groups for panels B and C utilize a two-sided Mann-Whitney U test.*

Fig. S17

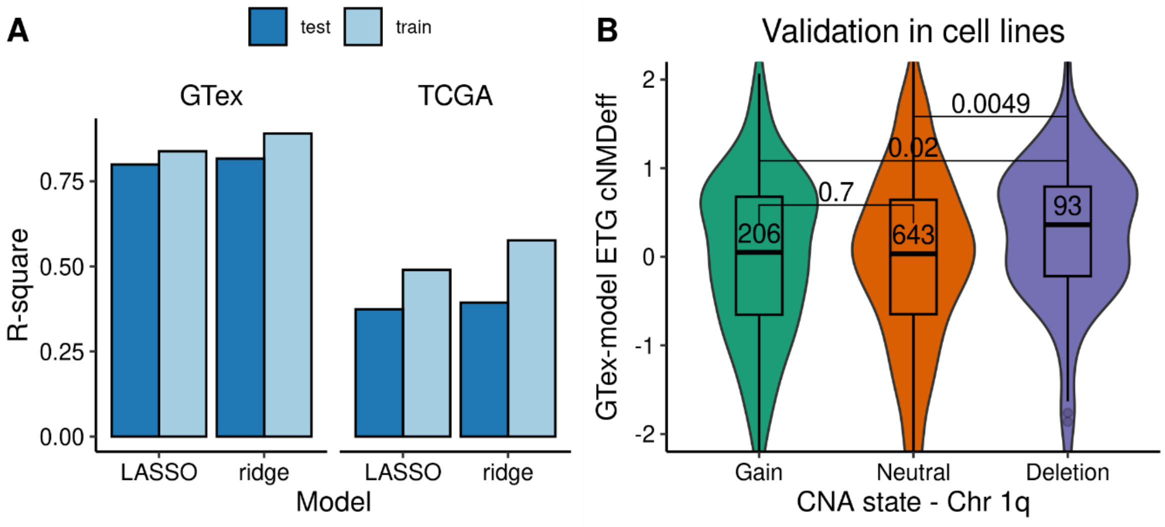

Fig. S17. Proxy-model of ETG iNMDeff using global gene-level expression data for validations in external datasets.

***A**, Predictive performance of the models to predict ETG iNMDeff using gene expression data, based on R-square values for LASSO and ridge regression models. These models are separately trained and tested within both GTex (left panel) and TCGA (right panel) cohorts. **B**, Validation of the negative impact of chromosome 1q gain on iNMDeff in 943 human cell lines (out of 1450 total ones) from the Cancer Cell Line Encyclopedia (CCLE). Here, a proxy-model, trained on GTex gene-level expression data, is applied to estimate cell line NMD efficiency (cNMDeff), categorized by their chr 1q arm status: gain, neutral, or deletion. Comparison between cell groups is conducted through a two-sided Mann-Whitney U test, underscore the statistical significance ($p < 0.05$) of 1q gain's effect on diminishing NMD efficiency.*

Fig. S18

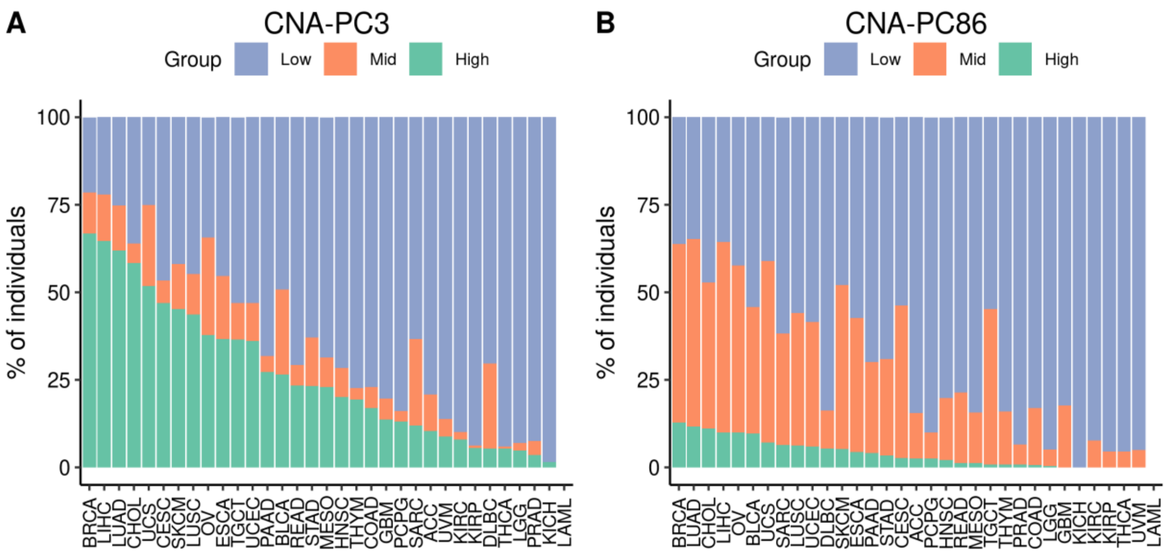

Fig. S18. Incidence of CNA-PCs 3 and 86 across cancer types.
A-B*, Distribution of CNA-PC groups (Low, Mid, High) shown as percentage of samples per cancer type, excluding LAML. Individual panels display distributions for CNA-PC 3 (A) and CNA-PC 86 (B).*

Fig. S19

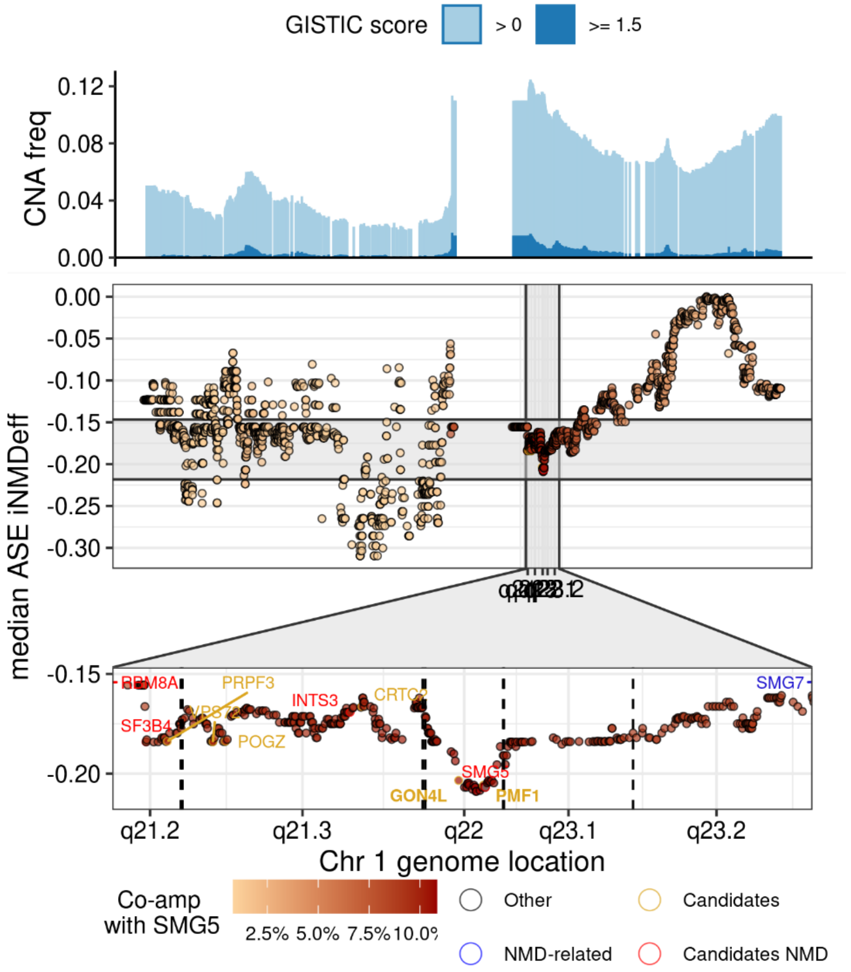

Fig. S19. Gene-level iNMDeff showcases how focal CNAs along chromosome 1q impact ASE NMD efficiency.

Pan-cancer CNA frequencies distinguishing high-confidence (GISTIC ≥1.5) and low-confidence (GISTIC >0) gene-level CNAs (top). The middle panel displays gene-level ASE iNMDeff scores, with red intensity indicating co-amplification frequency with NMD factor gene SMG5. These ASE iNMDeff scores are determined by the median value across individuals presenting focal CNAs within each gene. The bottom panel details a zoom view of 1q21.1-23.1 region highlighting iNMDeff reduction in amplified regions, with some selected genes categorized as Candidates, NMD-related (NMD genes outside the 1q21.1-23.1 region), Candidates NMD (NMD genes within the 1q21.1-23.1 region) or Other.

Fig. S20

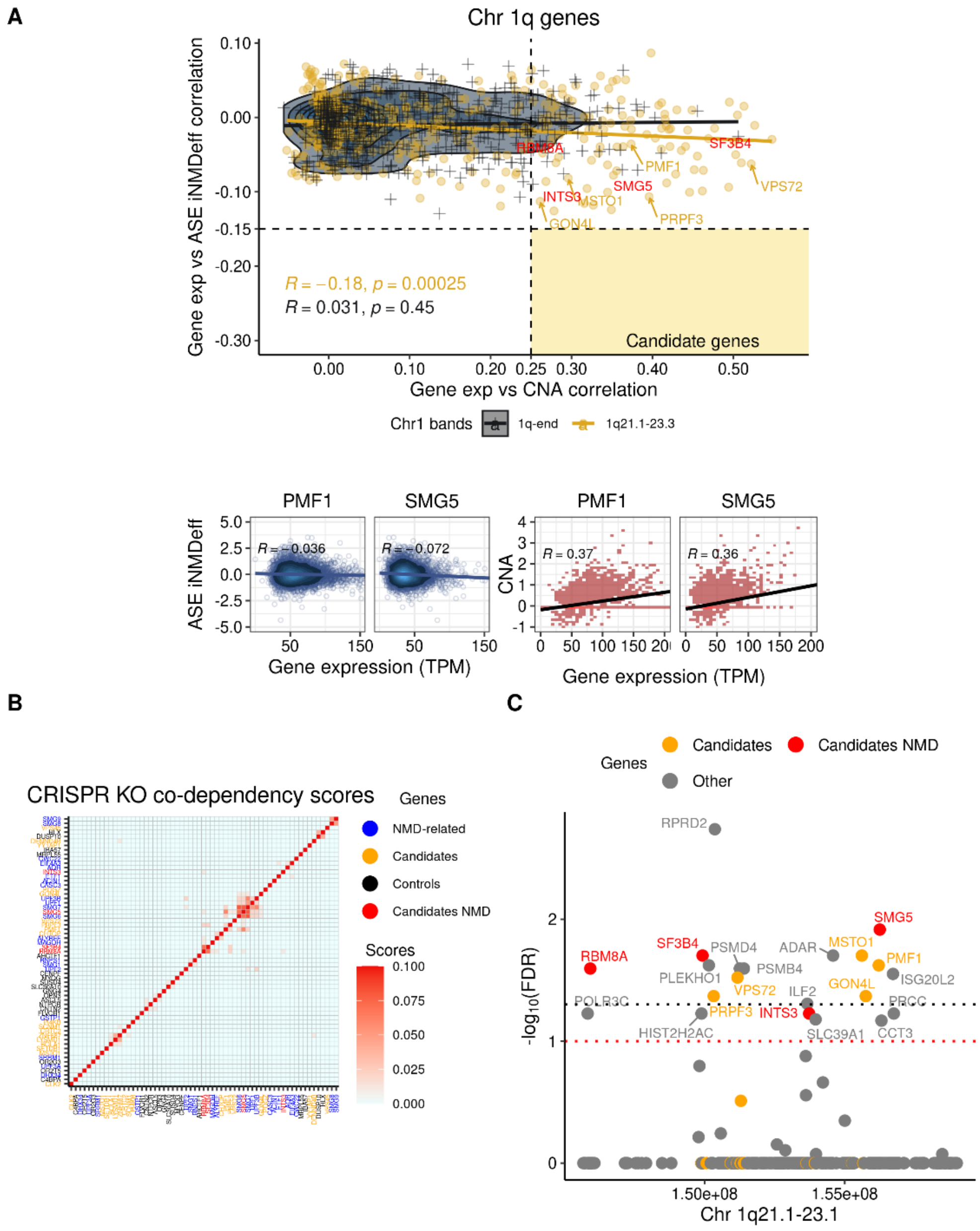

Fig. S20. Prioritization of candidate genes from the chromosomal arm 1q which exhibit CNA gains.

A, *Prioritization of candidate genes from chromosome 1q based on the correlation between gene expression (TPM) and ASE iNMDeff, juxtaposed against correlations between gene expression and CNA amplifications. Vertical and horizontal dashed black lines denote the thresholds for selecting candidates. The anticipated candidates reside in the quadrant underscoring a negative correlation between gene expression and ASE iNMDeff, alongside a positive correlation with CNA amplification. Genes within 1q21.1-23.1 are shown in orange, with NMD genes (SMG5, RBM8A, SF3B4, INTS3) highlighted in red, and remaining chromosome 1q genes in black. No candidates were found, but some of the candidates from the ETG iNMDeff analysis were highlighted instead. Two specific*

examples are shown below, which includes PMF1 and NMD-factor SMG5, showcasing their gene expression relative to ASE iNMDeff (bottom left) and CNA amplification (bottom right). ***B****, Clustering heatmap of CRISPR KO co-dependency scores of our four gene categories: 18 “Candidates” (orange), 4 “Candidates NMD” (red), 21 “NMD-related” genes (blue), 18 random “Controls” (black) from chr1q but outside of the 1q21.1-23.1 region. Of note, SF3B4 and RBM8A, despite not meeting ETG iNMDeff thresholds, were included as an additional “Candidates NMD” based on its related functions in NMD (spliceosome and EJC, respectively) and its location within 1q21.1-23.1. The normalization method, RPCO (onion), transforms the scores into an affinity matrix, where higher scores indicate a stronger genetic association.* ***C****, Test calculating the mean of CRISPR KO co-dependency scores, comparing all 274 genes from within 1q21.1-23.1 region with 10 core NMD factor genes versus 18 random control genes from the same chromosome but outside the 1q21.1-23.1 region. Genes with significant co-dependencies are highlighted, illustrated by -*log_{10}*(FDR) across two thresholds using horizontal dashed-lines: 5% (black) and 10% (red). P-values are obtained through a one-sided Mann-Whitney U test. X-axis represents the genome location.*

Fig. S21

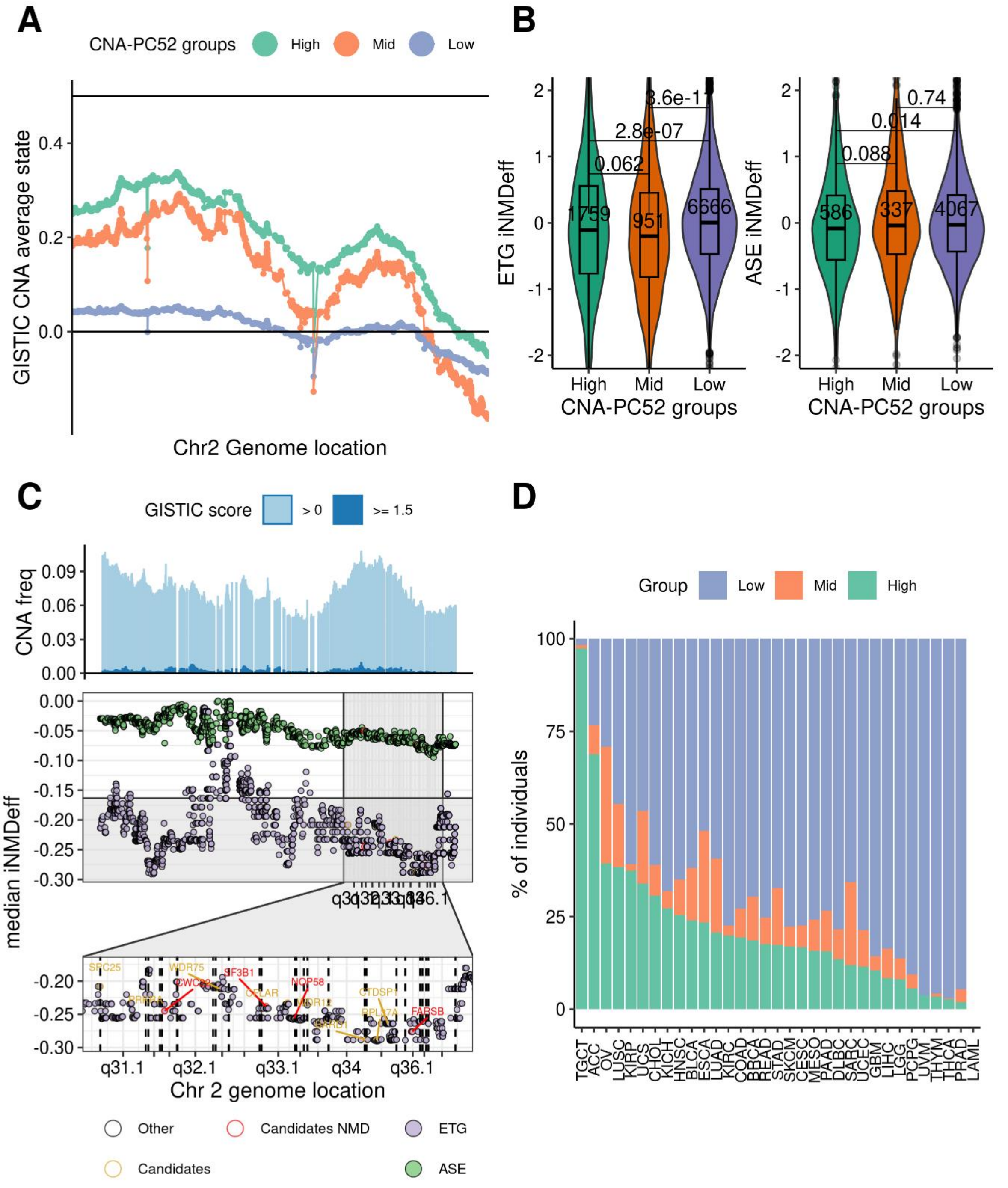

Fig. S21. Somatic chromosome 2q gain associates with reduced NMD efficiency.
A, *Gene-level amplifications across chromosome 2, ordered by genome location, assessed by averaging GISTIC CNA scores for each gene across TCGA participants. Samples are classified into three group bins based on their pan-cancer CNA-PC52 scores: "High", "Mid", and "Low".* ***B****, Stratification of scaled ETG (left panel) and ASE (right panel) iNMDeff according to these CNA-PC52 groups from A. Statistical comparisons between groups utilize a two-sided Mann-Whitney U test.* ***C****, Pan-cancer CNA frequencies distinguishing high-confidence (GISTIC ≥1.5) and low-confidence*

(GISTIC >0) gene-level CNAs (top). The middle panel displays gene-level ASE (green) and ETG (pink) iNMDeff scores. These iNMDeff scores are determined by the median value across individuals presenting focal CNAs within each gene. The bottom panel details a zoom view of the 2q31.1-2q36.3 region highlighting iNMDeff reduction in the amplified region, with genes categorized as "Candidates" (some are highlighted), "Candidates NMD" (NMD genes within the 1q21.1-23.1 region), and "Other". ***D**, Distribution of CNA-PC 52 groups (Low, Mid, High) shown as percentage of samples per cancer type, excluding LAML.*

Fig. S22

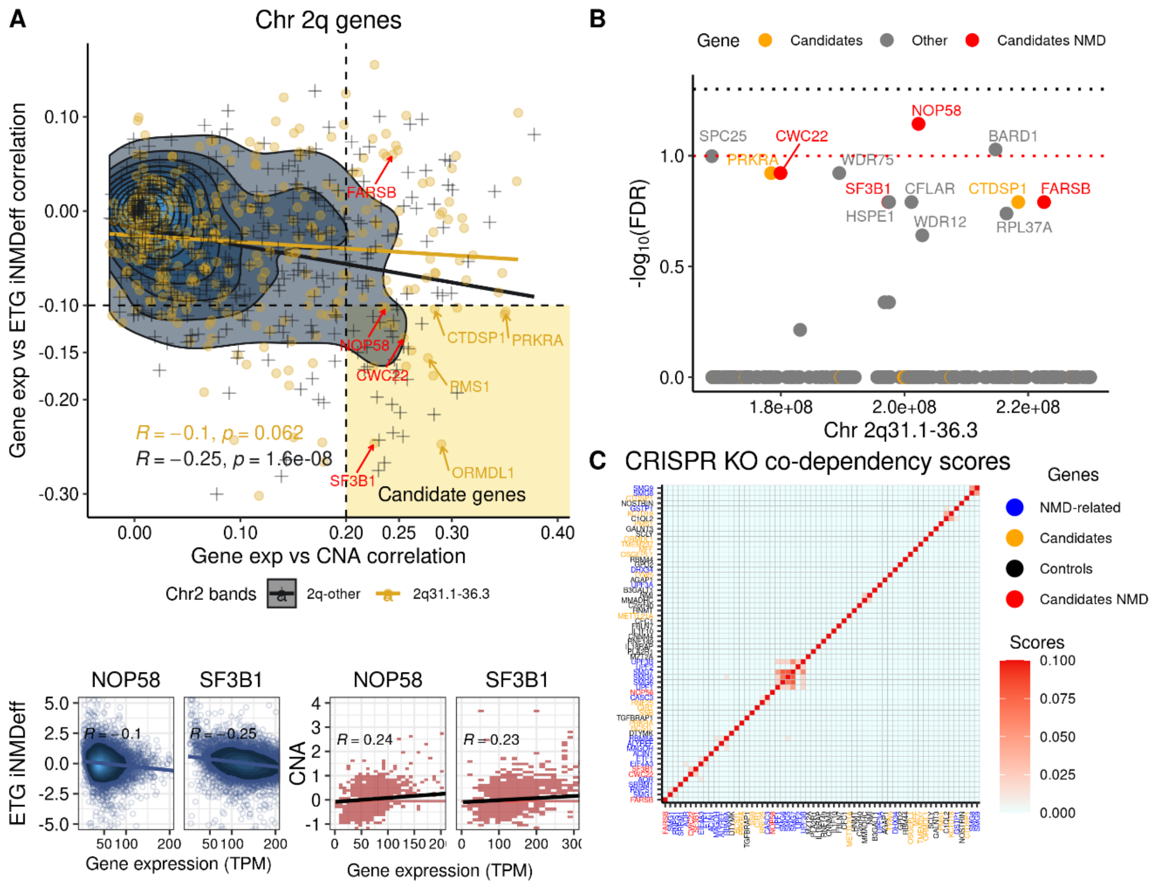

Fig. S22. Prioritization of candidate genes from the chromosomal arm 2q which exhibit CNA gains.

A, *Candidate gene prioritization from chromosome 1q using two scoring criteria: Correlation between gene expression and ETG iNMDeff (Y-axis) or CNA amplification (X-axis). Candidate genes are expected in the bottom-right quadrant, indicating sufficient correlation between expression and iNMDeff (negative) and between expression and CNA amplification (positive). Vertical and horizontal dashed black lines denote the thresholds for selecting candidates. Genes within 2q31.1-2q36.3 are shown in orange, with NMD-related genes (FARSB, SF3B1, CWC22, NOP58) highlighted in red, and remaining chromosome 2q genes in black. Of 45 genes meeting thresholds, 17 reside within the 2q31.1-2q36.3 region (14 "Candidates" and 3 "Candidates NMD" genes: SF3B1, CWC22 and NOP58), and 28 are from other 2q regions. Example candidate genes NOP58 and SF3B1 are displayed in the bottom panels, showing gene expression vs. ETG iNMDeff (bottom left) or CNA amplification (bottom right).* ***B***, *Test calculating the mean of CRISPR KO co-dependency scores, comparing all 303 genes from within 2q31.1-2q36.3 region with 10 core NMD factor genes versus 383 random control genes from the same chromosome but outside the specific region. Genes with significant co-dependencies are highlighted, illustrated by -log$_{10}$(FDR) across two thresholds using horizontal dashed-lines: 5% (black) and 10% (red). P-values are obtained through a one-sided Mann-Whitney U test. X-axis represents the genome location.* ***C***, *Clustering heatmap of CRISPR KO co-dependency scores for 14 "Candidates" (orange), 4 "Candidates NMD" (red; FARSB, SF3B1, CWC22, NOP58), 21 NMD-related genes (blue), and 22 random "Controls" (black) from chr2q,*

particularly focusing on the region 2q31.1-2q36.3. The normalization method, RPCO (onion), transforms the scores into an affinity matrix, where higher scores indicate a stronger genetic association. Of note; FARSB, despite not meeting ETG iNMDeff thresholds, was included as an additional "Candidates NMD" based on its related function with NMD and its location within 2q31.1-2q36.3 region.

Fig. S23

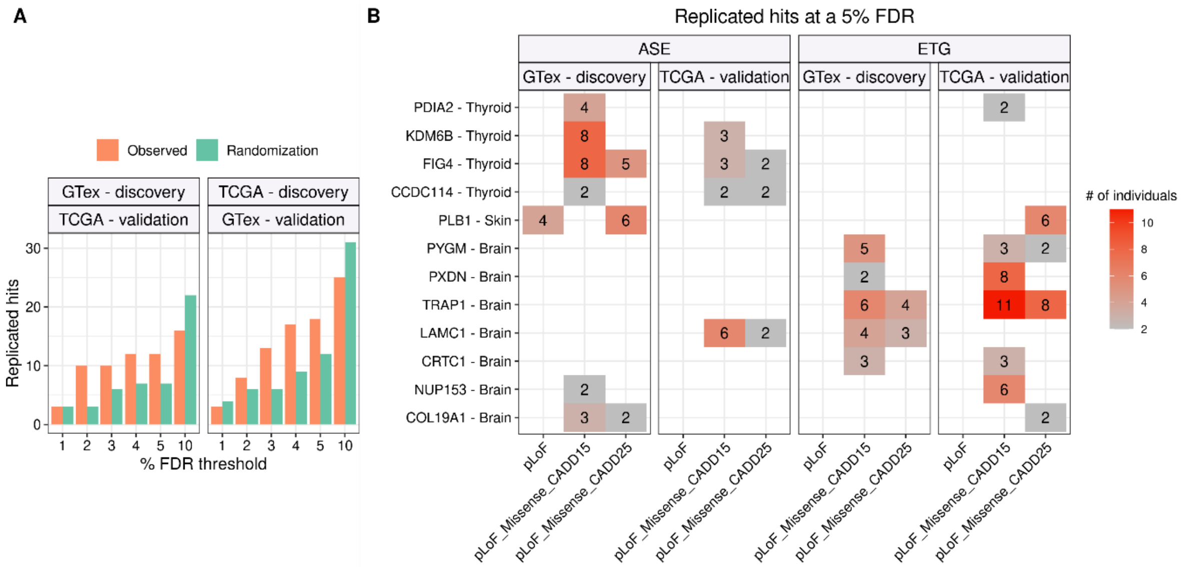

Fig. S23. Rare deleterious germline variants are associated with NMD efficiency.

A, *Number of replicated hits from our rare variant association analysis (RVAS), differentiated by the cohorts TCGA or GTex and whether the direction is from discovery to validation or vice versa on top panels, across varying FDR thresholds from 1 to 10%. The orange bars depict observed hits, while the green bars represent hits from randomizations of iNMDeff values, offering insights into the robustness of the associations against random expectations.* ***B,*** *Detailed view of replicated associations at 5% FDR. Shows replicated gene-tissue pairs at a 5% FDR threshold, categorized by putative loss-of-function (pLoF) variant set, NMD method, and the cohort where the association was identified (either discovery or validation). The values indicate the total count of individuals harboring a rare pLoF variant within the successfully replicated genes. Replication did not require the use of the same iNMDeff method as in the discovery cohort. For instance, for LAMC1 association, it was discovered in brain GTex using ETG method, and was further validated in brain tumors TCGA using ASE method.*

Fig. S24

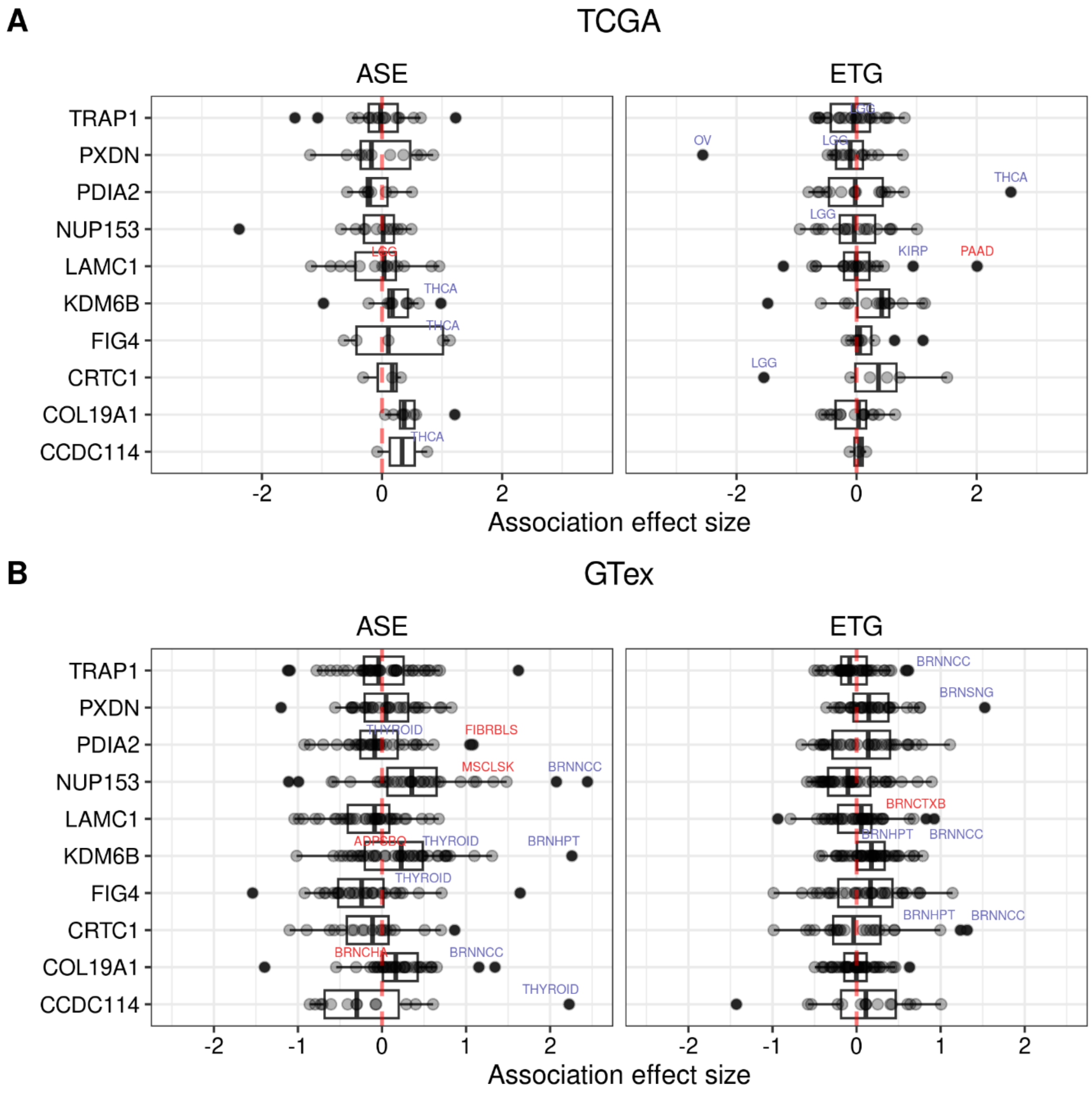

Fig. S24. Gene burden test associations of rare germline pLoF variants with iNMDeff for 10 replicated hits.

***A-B**, Linear model beta coefficients for significant hits plotted across cancer types (TCGA, A) and normal tissues (GTex, B), analyzed separately using ASE (left) and ETG (right) methods. Significant associations (SKAT-O FDR <2%, magenta) and suggestive associations (FDR <20%, red) are highlighted.*

Fig. S25

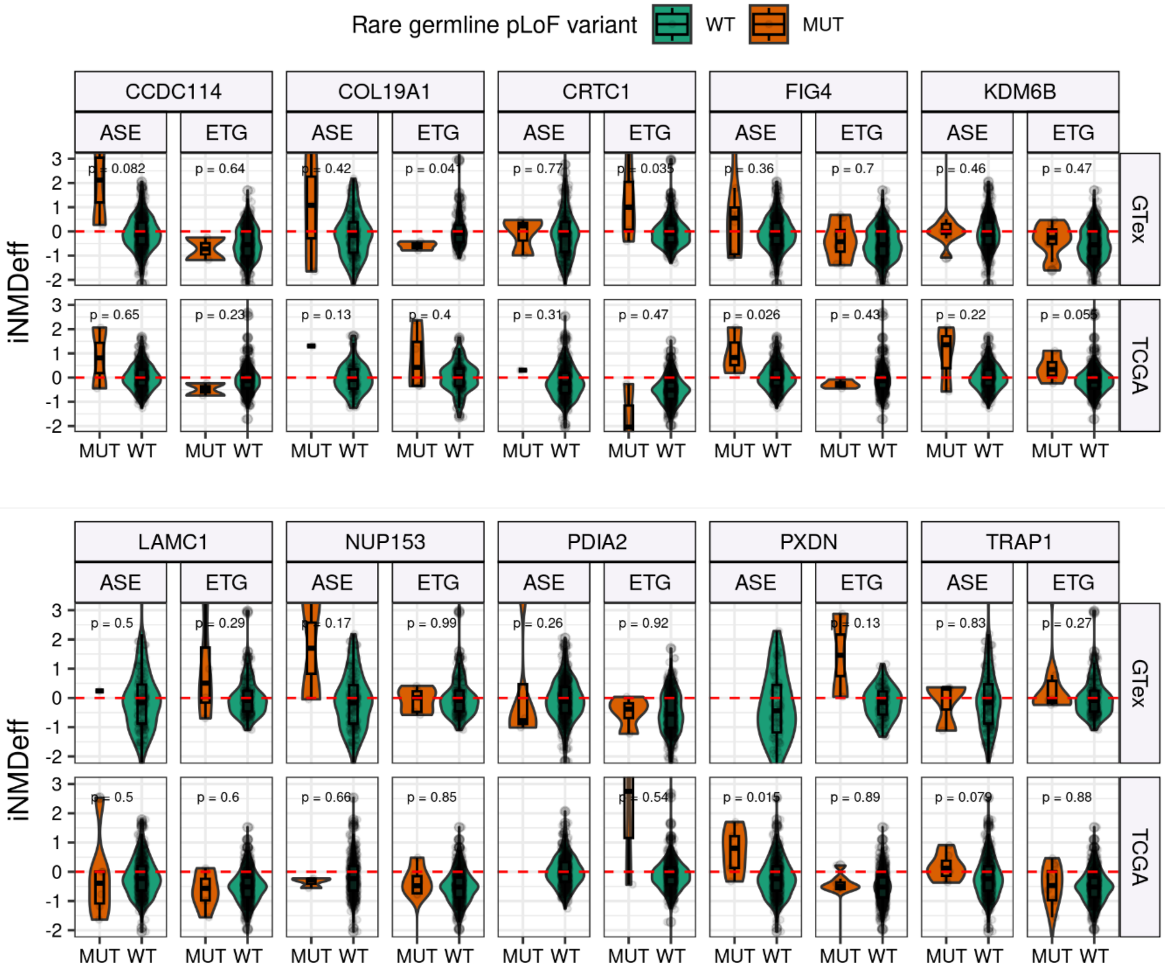

Fig. S25. iNMDeff of individuals with and without rare pLoFs on the 10 replicated hits from the RVAS analysis.

iNMDeff between individuals harboring wild-type (WT) alleles and those with rare pLoF mutations (MUT) within the 10 genes identified as significant in our RVAS analysis (SKAT-O FDR < 2%). These genes include CCDC114, COL19A1, CRTC1, FIG4, and KDM6B showcased in the top panels, and LAMC1, NUP153, PDIA2, PXDN, and TRAP1 depicted in the bottom panels. The analysis spans both TCGA and the GTex cohorts and is further distinguished by the method of NMD efficiency estimation, either ASE or ETG. Statistical significance between the WT and MUT groups is assessed using two-sided Mann-Whitney U tests, when applicable.

Fig. S26

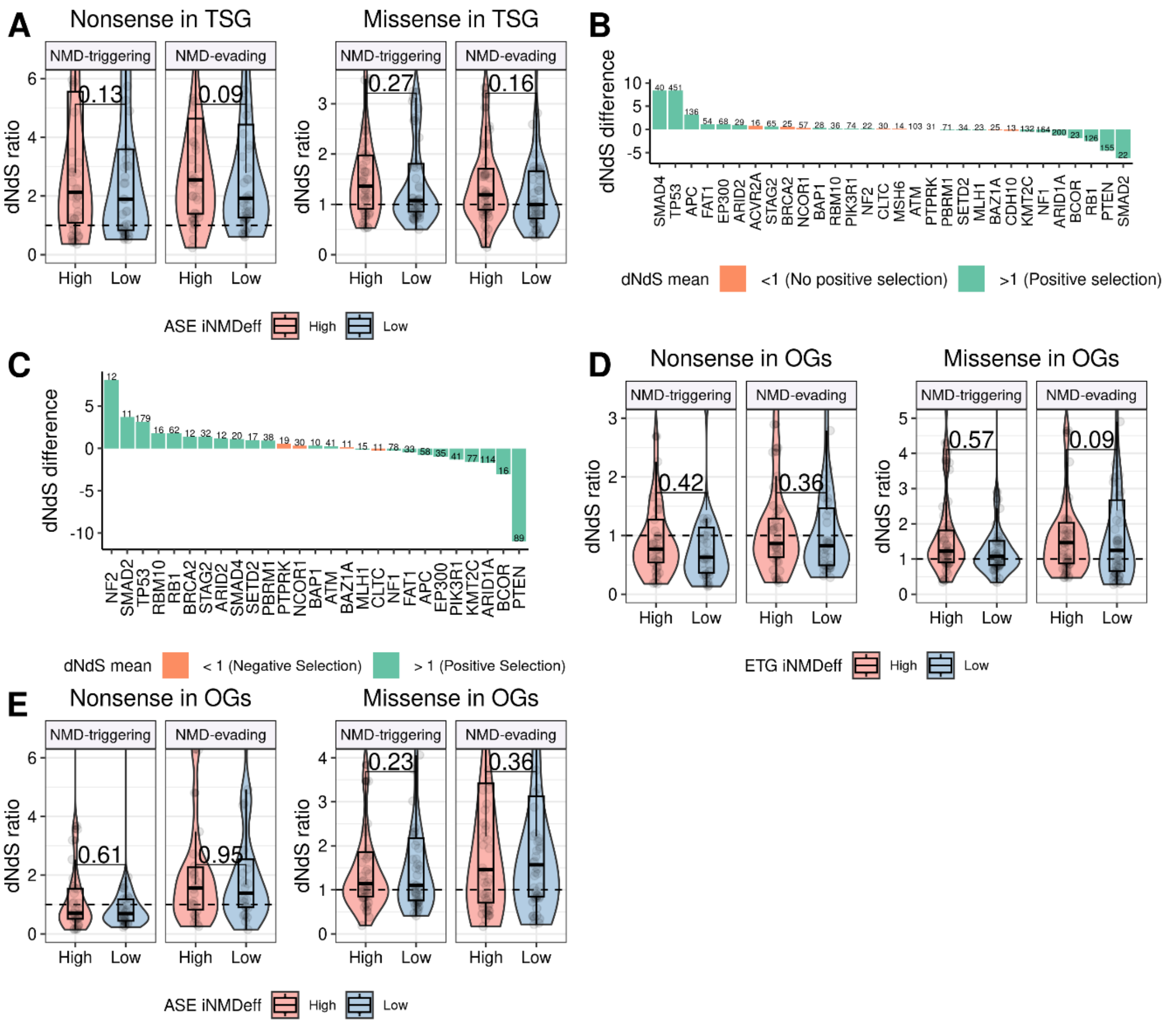

Fig. S26. Individual-level NMD efficiency modulates selection of somatic nonsense mutations.

***A**, dNdS ratios (via dNdScv method) of tumor suppressor genes (TSGs) for both NMD-triggering and NMD-evading nonsense (left panel) and missense (right panel) somatic mutations are compared between two groups of individuals with High (red) and Low (blue) iNMDeff, as determined by the median ASE iNMDeff. Statistical significance is assessed using one-sided Mann-Whitney tests on paired data. PTCs were classified as NMD-triggering if they met all criteria: (1) >250nt from TSS, (2) in exons ≤500nt, and (3) not in the last exon or last 55nt of the penultimate exon. Conversely, PTCs were classified as NMD-evading if they met any criteria: (1) ≤250nt from TSS, (2) in exons ≥1000nt, or (3) in the last exon or last 55nt of the penultimate exon. **B,** The differences in dN/dS between high and low ETG iNMDeff groups are plotted for each gene, specifically for NMD-triggering nonsense mutations within TSGs. Positive values indicate a stronger selection pressure in the high iNMDeff group compared to the low group, and vice versa. The number above each bar denotes the total count of nonsense mutations contributing to the dN/dS calculation for both groups. Genes with less than 10 counts were removed. The barplot colors correspond to the mean dN/dS ratios of the two iNMDeff groups (high and low): green indicates a mean ratio greater than 1, suggesting positive*

selection, while orange signifies a mean ratio less than 1, indicative of lack of positive selection. ***C****, Same as B but for differences in dN/dS ratios between High and Low ASE iNMDeff groups of samples.* ***D-E****, Same as A but for oncogenes (OGs) using either ETG (D) or ASE (E) iNMDeff individuals.*

Fig. S27

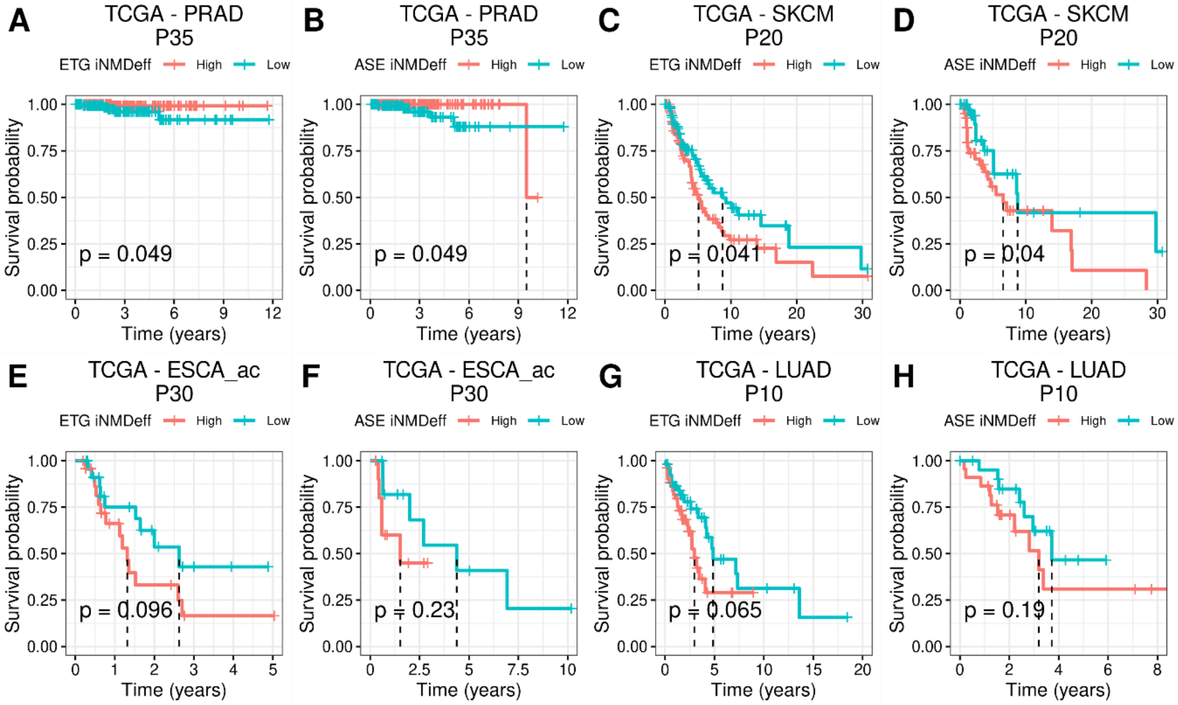

Fig. S27. Individual-level NMD efficiency impacts overall survival (OS) in diverse cancer types.

A-H, *Kaplan-Meier (KM) survival curves comparing overall survival (OS) outcomes between groups with High (red) versus Low (blue) iNMDeff, as determined by the median of ASE or ETG iNMDeff in TCGA cancer types: prostate adenocarcinoma (PRAD; percentile 35th) (A-B), skin cutaneous melanoma (SKCM; percentile 20th) (C-D), esophageal squamous-cell carcinoma (ESCA_acc; percentile 30th) (E-F) and lung adenocarcinoma (LUAD; percentile 10th) (G-H). Log-rank test p-values quantify the statistical significance of the survival differences for all KM curves (A-H)*

Fig. S28

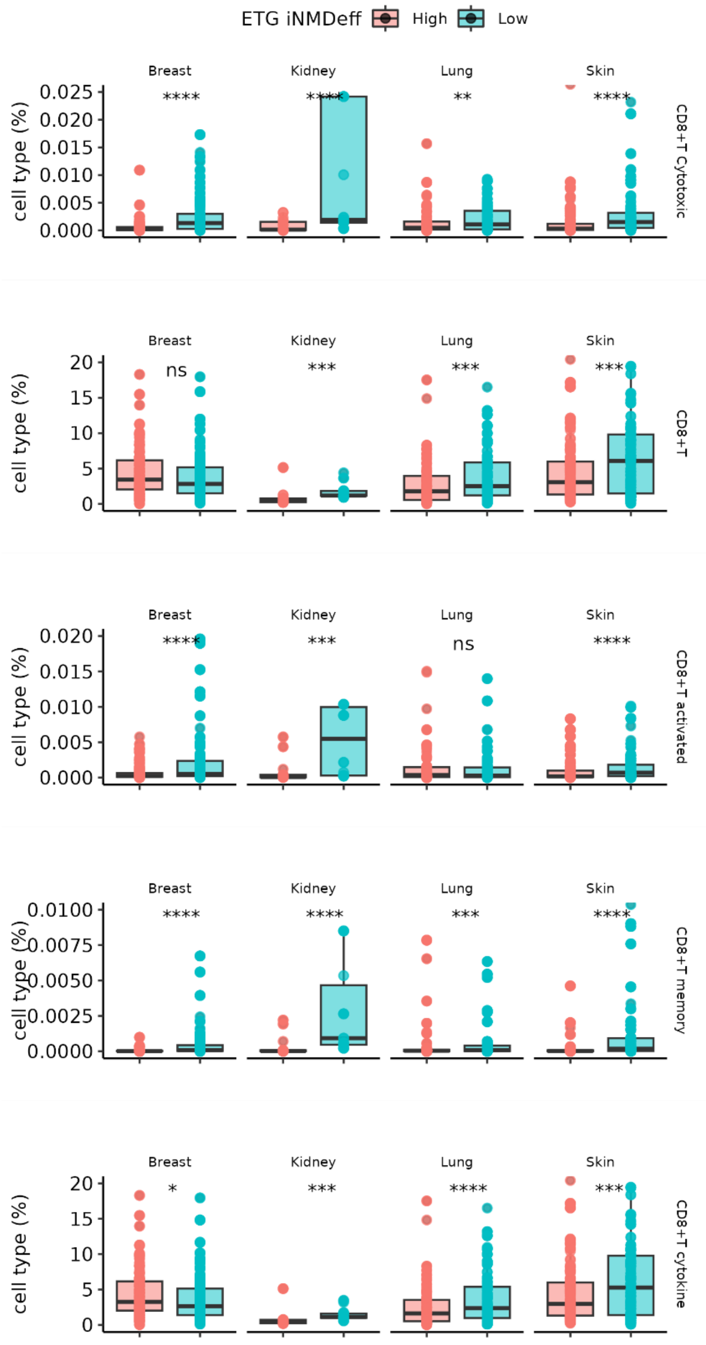

Fig. S28. Tumors with reduced NMD efficiency correlate with increased CD8+ T-cell infiltration.

CD8+ T-cell subset infiltration proportions in the Hartwig cohort comparing tumors with high versus low NMD efficiency (top 30% vs bottom 30% ETG iNMDeff estimates from GTex-based proxy model) across melanoma, lung, kidney, and breast cancers, for patients treated by either immunotherapy or chemotherapy. Immune cell proportions were estimated from RNA-seq data using UCD Base for five CD8+ T-cell subsets: Cytotoxic, general CD8+ T, Activated, Memory, and Cytokine-secreting effector cells.

Fig. S29

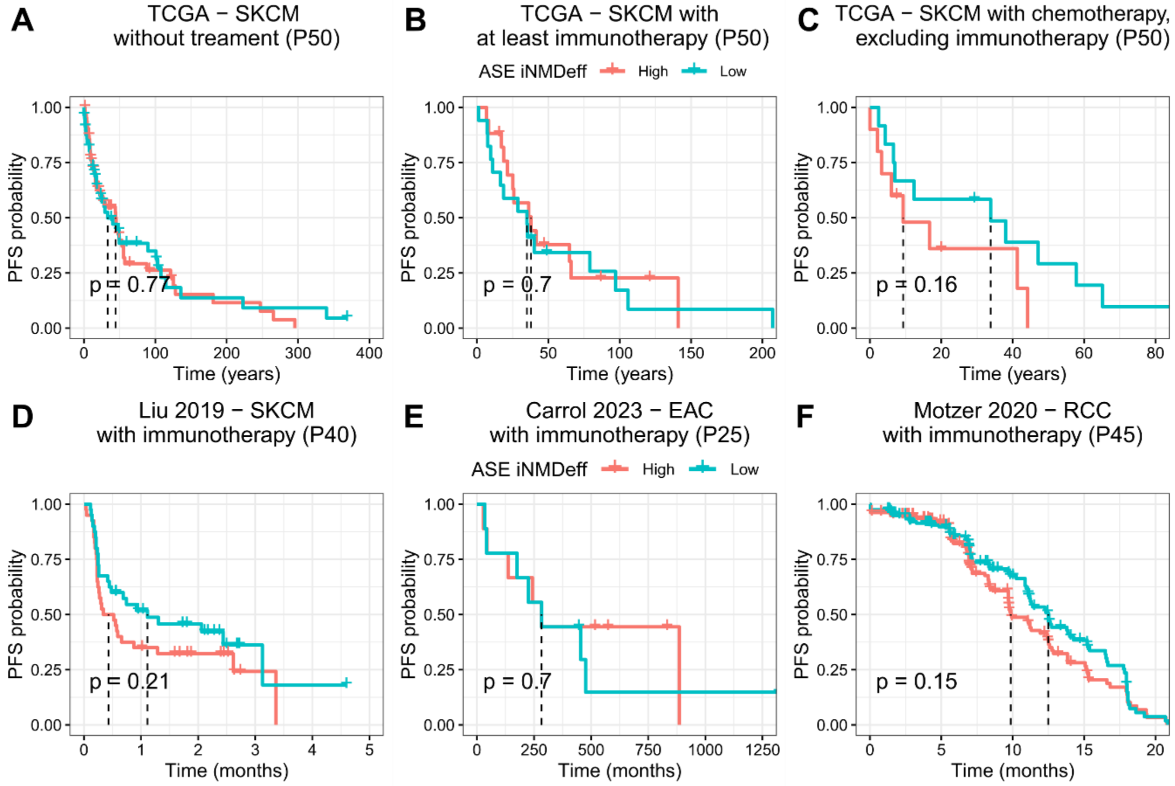

Fig. S29. NMD efficiency impacts progression-free survival (PFS) and response to immunotherapy in treated patients

A-C, *Kaplan-Meier (KM) survival curves comparing progression-free survival (PFS) outcomes between groups with High (red) versus Low (blue) iNMDeff, as determined by the median of ASE iNMDeff in TCGA SKCM. The analysis is divided into three patient categories: no treatment data available (A), those treated with immunotherapy at least once (B), and those treated exclusively with chemotherapy, excluding immunotherapy patients (C). Log-rank test p-values quantify the statistical significance of the survival differences.* ***D-F,*** *Validation of the KM curves for PFS in external patient cohorts from Liu et. al. 2019 (SKCM – skin cutaneous melanoma, D) at the 40th percentile, Carrol et. al. 2023 (EAC - esophageal adenocarcinoma, E) at the 25th percentile, and Motzer et. al. 2020 (RCC - renal cell carcinoma, F) at the 45th percentile. Patients treated with Sunitinib were excluded from this analysis.*
