# Supplementary material for: Variable efficiency of nonsense-mediated mRNA decay across human tissues, tumors and individuals
Source: Genome Biol. 2025 Sep 29;26:316. doi: 10.1186/s13059-025-03727-y (PMC12477815; doi:10.1186/s13059-025-03727-y)
Supplement: Supplementary file 3 — Additional File 3: Supplementary Text S1-S6 [file 13059_2025_3727_MOESM3_ESM.pdf]

Supplementary Text S1-S6 for Palou and Supek (2025) *Genome Biology*.

Text S1: Additional support for robustness of NMD efficiency measures
Text S2: Optimizing sample size and precision in ASE-based NMD efficiency estimation
Text S3: Inter-individual NMD efficiency variability
Text S4: Association analysis of somatic mutations with iNMDeff
Text S5: Somatic chromosome 2q gain also associates with reduced NMD efficiency
Text S6: NMD efficiency modulates selection of somatic nonsense mutations

Text S1: Additional support for robustness of NMD efficiency measures

In TCGA and GTex, the iNMDeff estimates from the ETG method (Fig. 2A and Additional File 1: Fig. S2A-B) and from the ASE method (Fig. 2B and Additional File 1: Fig. S2C) show various trends that imply they are reliable. All the ETG NMD gene sets from the sourced studies showed consistent efficiency scores across studies, with "NMD Tani" displaying notably higher scores. This difference may be attributed to its unique use of RefSeq annotation rather than Ensembl, potentially capturing different NMD-triggering features, particularly in the UTR regions. Unlike RefSeq, which only contains protein-coding genes, Ensembl specifically includes NMD decay transcripts in its annotation. The higher-confidence "NMD Consensus" ETG gene set displayed a readout of slightly higher efficiency (i.e. the fold-difference in expression between the NMD transcript and its non-NMD counterpart in the same gene) than the more permissive "NMD All", which presumably contains weaker NMD target genes (TCGA: $p < 2e-16$, Mann-Whitney U test). The negative control gene set "RandomGenes without NMD features" shows negligible efficiency (TCGA: $p < 2e-16$, when compared to "NMD Consensus"), with the median close to zero (Fig. 2A). Underscoring the reliability of the features we used to identify NMD-targeted transcripts for the ETG method, the gene set "RandomGenes with NMD features" exhibits efficiency similar to the experimentally identified NMD gene sets, with a very slight albeit significant difference (median iNMDeff = 2.10 vs 2.11, $p = 4.8e-2$, when comparing to "NMD Consensus"). This result also suggests the presence of many additional genes with at least one NMD-targeted transcript not identified experimentally in studies considered here[5,6,13,51]. In GTex, we saw very similar results as described above (Additional File 1: Fig. S2B-C).

In an additional analysis, we ruled out differential transcription rates as a confounding factor in our estimation of NMD efficiency from mRNA levels. We analyzed PRO-seq data, which captures nascent RNA transcripts, from three cell lines (A549[56], U2OS[56], and SH-SY5Y[55]). It provides nucleotide-resolution measurements of active RNA polymerase II transcription across the genome. We compared promoter activity between NMD-target and non-target transcripts from our ETG Consensus set by measuring PRO-seq signals near the TSS and downstream regions (see Methods). No significant differences in transcription rates were observed between NMD and non-NMD transcripts, regardless of cell type (lung A549, bone U2OS, or neural SH-SY5Y) (Additional File 1: Fig. S3B). To specifically address brain-associated differential NMD efficiency, we stratified transcripts into brain-upregulated and non-brain-upregulated categories based on transcript expression from GTex and TCGA datasets and we observed no significant differences. These findings confirm that differences in transcript levels in our ETG measure reflect NMD activity rather than transcriptional variation.

We also tested correlations between ASE iNMDeff and ETG iNMDeff and other biological covariates or technical variables, including all our NMD gene/variant sets: age, sex, tumor mutation burden (TMB), tumor indel burden (TIB), tumor nonsense burden (TNB), purity, MSI score, RNA-seq sample library size, leukocyte fraction, CNA burden, and number of NMD targets used to estimate iNMDeff (either PTCs for ASE, or transcripts for ETG) (Additional File 1: Fig. S6B-C). None of the tested covariates did show strong correlations with any of our iNMDeff in either TCGA or Gtex. For those that had a small effect (CNA burden R = -0.1 in TCGA; RNA-seq sample library size R = -0.13-16; age R = -0.15 in GTex), we included them always in our downstream association analysis as covariates. The "NMD Consensus" gene set correlates the highest to ASE iNMDeff (Additional File 1: Fig. S4A-B and S6B-C), and thus we used it as the default gene set for the ETG method (list of transcripts in Additional File 2: Table S4). Also the negative control gene/variant sets (non-NMD target genes; synonymous mutations and NMD-evading PTCs) did not exhibit correlations with our iNMDeff estimates. This suggests there were not strong confounders that change expression of many genes in trans (for ETG: R = -0.04-0.03 between "NMD Consensus" and "RandomGenes without NMD-triggering features"; for ASE: R = 0.04-0.15 between "NMD-triggering PTCs" and "NMD-evading PTCs" or "Synonymous") (Additional File 1: Fig. S6B-C). Indeed, the ASE method, which measures allele-specific expression of PTCs, is widely used to monitor NMD efficiency of PTCs[8,10,143,161]; ASE approach is robust against confounding factors since they are likely to affect both alleles similarly.

Text S2: Optimizing sample size and precision in ASE-based NMD efficiency estimation

Our ASE method shows lower precision compared to ETG due to its dependence on germline PTC variants. While ETG leverages expression data from ~100 NMD genes, ASE relies on the limited number of PTC variants present in each genome -- which means that the readouts for the few-PTC genomes are noisier than the readouts from the many-PTC genomes. While measurement noise can be reduced by requiring more PTCs per sample, this creates a trade-off between precision and sample retention for downstream analyses.

Our current analysis implemented a minimum threshold of ≥3 PTCs per sample, which excluded 47% of TCGA and 25% of GTex samples. To evaluate the impact of this threshold choice, we tested requirements ranging from 1-10 PTCs, comparing pan-tissue/cancer ASE-ETG Pearson correlations (R) across thresholds. Increasing from 3 to 6 PTCs improved correlations from ~0.14-0.19 to ~0.23 in both cohorts (Additional File 1: Fig. S5A, upper panels). This improvement was consistent across tissue types (Additional File 1: Fig. S5B, upper panels): in GTex, the proportion of tissues showing positive correlations increased from 75% to 86%, while in TCGA, the mean tissue-specific correlation improved from 0.09 to 0.19. However, this stricter threshold would have excluded 85% of TCGA and 65% of GTex samples. This analysis also controlled for the varying sample sizes across different thresholds by a subsampling to generate a matched-sample size baseline for each threshold.

We also examined the effect of population allele frequency (AF) filtering. Our initial analysis used AF ≤ 0.2 to exclude very common PTCs due to them being under selection, which results in constraints on NMD efficiency[8,143]. When applying a stricter filter of AF ≤ 0.01 and obtaining a smaller but less noisy dataset, ASE-ETG pan-tissue/cancer correlations improved

substantially (Additional File 1: Fig. S5A, bottom panels): GTex correlations reached 0.24-0.70 and TCGA correlations reached 0.28-0.97 (for 5-10 PTC thresholds). By tissue correlations also improved greatly, where the mean of correlation across tissues increased from ~0.1 to ~0.6 (Additional File 1: Fig. S5B, bottom panels). We note that pushing the ASE-ETG correlations to values of >=0.70 would have required drastic reductions in the number of available samples (Additional File 1: Fig. S5).

Given these trade-offs, we selected final parameters (≥3 PTCs, AF ≤ 0.2) that balanced measurement precision with statistical power for subsequent association analyses, for example, with CNAs and germline rare variants. This choice represents a compromise between optimal ASE-ETG correlation and retention of sufficient samples for downstream statistical analyses. We reasoned that statistical power is a better strategy, particularly given that ASE is used largely as a replication method i.e. no conclusions are made on ASE alone but ETG support is required.

Text S3: Inter-individual NMD efficiency variability

Using our Tissue-wise iNMDeff Deviation (TND) test, we checked tissue variability in NMD efficiency, where positive TND values indicate that a tissue exhibits higher iNMDeff than what would be expected by chance, and vice versa for negative values (subset in Fig. 3C-D, complete set in Additional File 1: Fig. S9A-B). Here, we comment on additional tissues/cancers with interesting iNMDeff differences.

Pan-reproductive tissues: Other normal Pan-reproductive tissues like vagina, testis, uterus, and prostate (PRSTTE) showed strong significant differences in iNMDeff in ETG and ASE methods in GTex. These differences were not significant in their respective cancer types in TCGA, though they generally followed the same downward trend, with exception of prostate. Interestingly, uterine corpus endometrial carcinoma MSI subtype (UCEC_MSI) showed significantly higher ETG iNMDeff (TND = 0.09, p = 1.23e-3), and higher than the MSS subtype (Additional File 1: Fig S9C-D), paralleling the MSI trends observed previously in COAD and STAD.

Pan-squamous tissues: Analysis of Pan-squamous tissues overall did not yield significant and/or consistent results across the two NMD methods, in either TCGA tumors or GTex tissues. Considering lung cancer specifically (LUSC and lung adenocarcinoma, LUAD), there is no consistency between ETG and ASE NMD estimation methods. Bladder urothelial carcinoma (BLCA) subtypes showed minimal variation in NMD efficiency: Basal squamous cell (BLCA_Basal_scc) and luminal infiltrated (BLCA_Lum_inf) subtypes showed no significant differences, while luminal papillary (BLCA_Lum_pap) exhibited lower iNMDeff in ETG (TND = -0.05, p = 1.7e-2) but not in ASE (TND = -0.01, p = 4.7e-1). In head and neck squamous cell carcinoma, human papilloma virus (HPV) positive tumors (HNSC_HPV_pos) showed higher iNMDeff in both methods (ETG: TND = 0.09, p = 4.9e-2; ASE: TND = 0.22, p = 1.28e-1), while HPV-negative tumors (HNSC_HPV_neg) showed less consistency between methods (ETG: TND = 0.18, p = 1.23e-3; ASE: TND = 0.02, p = 0.47). Notably, our observation of higher NMD efficiency in HPV-positive tumors contrasts with previous findings that HPV inhibits NMD activity[162].

Pan-kidney tissues: Among Pan-kidney tissues, only kidney renal clear cell carcinoma (KIRC) showed significant differences in NMD efficiency, specifically in the ASE method (TND = 0.13, p = 8e-3). This finding aligned with normal kidney tissues, where both cortex (KDNCTX: TND = 0.11, p = 4.9e-2) and medulla (KDNMDL: TND = 0.52, p = 3.3e-2, n = 4) showed also higher NMD efficiency. Kidney renal papillary cell carcinoma (KIRP) showed no significant differences.

Other tissues: Among tissues and cancers not classified into 'pan' categories (Additional File 1: Fig. S9A-B), we observed several notable patterns in NMD efficiency.

Most strikingly, we found contrasting patterns between normal tissues and their corresponding cancers. Normal pancreatic tissue (PNCREAS) and whole blood (WHLBLD) showed higher NMD efficiency (ETG: TND = 0.04-0.14, p = 8.7e-4; ETG: TND = 0.14, p = 8.7e-04 and ASE 0.05, p = 2.5e-2, respectively) compared to their respective cancers - pancreatic adenocarcinoma (PAAD, ETG: TND = -0.1, p = 1.2e-3) and acute myeloid leukemia (LAML, ETG: TND = -0.08, p = 1.2e-3; ASE: TND = -0.29, p = 5.9e-3). Similarly, normal muscle tissue (MSCLSK) exhibited higher iNMDeff (ETG: TND = 0.1, p = 8.7e-4; ASE: TND = 0.1, p = 2.5e-3), compared to both muscle (SARC_Muscle, ETG: TND = -0.14, p = 1.2e-3; ASE: TND = -0.04, p = 4.7e-1) and fat sarcomas (SARC_Fat, ETG: TND = -0.08, p = 1.7e-2). Conversely, liver hepatocellular carcinoma (LIHC) showed higher NMD efficiency (ETG: TND = 0.03, p = 1.7e-2; ASE: TND = 0.23, p = 5.9e-3) than normal liver tissue (ETG: TND = -0.07, p = 8.7e-4).

However, we also observed tissues where both normal and cancer samples showed similar trends. For instance, both adrenocortical carcinoma (ACC) and normal adrenal gland (ADRNLG) displayed higher iNMDeff (ETG: TND = 0.07, p = 1.6e-2; ETG: TND = 0.22, p = 8.7e-04 and ASE 0.05, p = 6.2e-2, respectively), as did sun-exposed skin (SKINS) and melanoma (SKCM) (ASE: TND = 0.04, p = 4.3e-2; ETG: TND = 0.10, p = 1.2e-3, respectively). Conversely, both normal thyroid tissue and thyroid cancer (THCA) showed consistently lower iNMDeff (ETG: TND = -0.11, p = 8.7e-4 and ASE: TND = -0.02, p = 1.2e-1; ETG: TND = -0.03, p = 1.6e-2, respectively).

Several cancers with no matched normal tissue displayed consistent patterns across both ETG and ASE methods. Kidney chromophobe (KICH, ETG: TND = -0.08, p = 1.7e-2 and ASE: TND = -0.1, p = 3.3e-1), cholangiocarcinoma (CHOL, ETG: TND = -0.16, p = 1.2e-3 and ASE: TND = -0.18, p = 2.4e-1), and thymoma (THYM, ETG: TND = -0.27, p = 1.2e-3) all exhibited lower iNMDeff.

In normal tissues with no matched cancer type, we identified several distinct patterns. Spleen and pituitary (PTTARY) showed lower NMD efficiency (ETG: TND = -0.08 to -0.19, p = 8.7e-4 and ASE: TND = -0.09 to -0.12, p = 2.5e-3 for both), while cardiovascular tissues consistently displayed higher iNMDeff, including various arteries (aorta –ARTAORT–, coronary –ARTCRN–, and tibial– ARTTBL; ETG: TND = 0.01 to 0.04, p = 1.3e-1 to 4.9e-3 and ASE: TND = 0.03 to 0.05, p = 1.3e-1 to 7.8e-2) and heart tissues (atrial appendage –HRTAA– and left ventricle–HRTLV; ETG: TND = 0.15 to 0.24, p = 8.7e-4 and ASE: TND = 0.06 to 0.09, p = 2.5e-3 to 2.4e-2). Both subcutaneous (ADPSBQ) and visceral omentum (ADPVSC) adipose tissues also showed higher ETG iNMDeff (TND = 0.03 to 0.08, p = 1.7e-3 to 8.7e-4). Notably, EBV-transformed lymphocytes (LCL) and cultured fibroblasts (FIBRBLS) showed extremely

high iNMDeff (ETG: TND = 0.31 to 0.41, p = 8.7e-4 and ASE: TND = 0.13 to 0.16, p = 6.75e-3 to 2.5e-3), though these findings should be interpreted cautiously due to limited sample sizes.

The remaining tissues and cancers either showed non-significant differences or demonstrated inconsistent patterns between NMD efficiency methods and TCGA-GTex cohorts.

Text S4: Association analysis of somatic mutations with iNMDeff

We hypothesized that genetic variants might generate the observed inter-individual variation in NMD efficiency (Additional File 1: Fig. S11C-D). We initiated our investigation by considering TCGA tumors, and testing for genetic associations between our iNMDeff estimates and somatic mutations. We adjusted for various covariates, and carried out the analysis for each gene and each cancer type individually, including additionally a pan-cancer analysis (as detailed in Methods). Our focus was on: i) a set of 727 recognized cancer driver genes from the Cancer Gene Census (CGC)[151]; ii) 112 genes related to the NMD pathway sourced from two experimental studies[69,70] and additional literature[3,7,150] (see Methods and Additional File 2: Table S9); iii) the remaining 18,780 genes were considered as a baseline ("random genes") for our analysis. We then tested these genes against different sets of somatic variants: firstly the point mutations -- synonymous, truncating (which includes nonsense, indels, and splicing variants), missense -- and secondly the CNAs.

For both analysis, in total we systematically conducted 6,493,740 tests across 19,619 genes and 33 cancer types (here, not stratified by subtype, to prevent increasing number of tests and reducing sample sizes), where each test utilized one NMD method (ASE or ETG) for discovery and the other for validation. To ensure the robustness of our association results, we calculated the lambda (inflation factor) for each cancer and type of somatic variant, excluding the few tests with lambda >1.5 (Additional File 1: Fig. S12A-B). All significant hits using one NMD estimation method in one particular cancer type were re-tested in the same cancer type with the other NMD method, adjusting by FDR within those hits. With this criteria, two replicated significant associations emerged at a FDR threshold of 5%: a missense variant in the *TLX1* gene in LUAD and a truncating variant in the *CDH1* gene in BRCA, both associated with iNMDeff with opposite directions (Additional File 1: Fig. S12C-D). Further exploration of the effect sizes of these two associations in other cancer types revealed that, for the *CDH1* association (mean ASE-ETG effect size = -0.15), the directionality was consistent, but not significant, in pan-cancer (effect size = -0.1) and UCEC (effect size = -0.09), both being negatively associated with iNMDeff (Additional File 1: Fig. S12E). For the *TLX1* association (effect size = 1.1), a consistent positive direction was observed in LUSC (0.34), Bladder urothelial carcinoma, BLCA (0.23), and Thyroid carcinoma, THCA (0.1), with a modest agreement also in pan-cancer (0.03), with associations in the opposite direction for other tumor types (Additional File 1: Fig. S12E). This supports the association of somatic mutations in the *TLX1* transcription factor with enhanced NMD efficiency.

Text S5: Somatic chromosome 2q gain also associates with reduced NMD efficiency

In our analysis of somatic copy number alterations, we identified CNA-PC52 as another pan-cancer principal component signature that showed a significant negative association with NMD efficiency. Individuals with higher scores in the CNA-PC52 signature exhibited CNA gain peaks at chromosome 2p and 2q, (Additional File 1: Fig. S21A), however, of relatively lower intensity (average GISTIC scores around ~0.2), suggesting this CNA-PC captures low-level gains. A decrease in iNMDeff was observed in individuals with the 2q gain peak spanning approximately the 2q31.1-2q36.3 region (Additional File 1: Fig. S21B-C). This CNA signature was prevalent in testicular germ cell tumors (TGCT, 97% of samples), adrenocortical carcinoma (ACC, 69%), OV (39%), LUSC (38%), and kidney renal papillary cell carcinoma (KIRP, 37%) (Additional File 1: Fig. S21D). Within this region, four NMD-related genes are located: *CWC22* (2q31.3), *SF3B1* (2q33.1), *NOP58* (2q33.1), and *FARSB* (2q36.1) (Additional File 1: Fig. S21C). Further investigation by our iNMDeff-gene expression and CNA-gene expression correlations identified 14 ”Candidates” genes and 3 “Candidates NMD” (including *CWC22*, *SF3B1* and *NOP58*) (Additional File 1: Fig. S22A).

Additionally, we analyzed the CRISPR KO codependency scores to assess whether any of the 303 genes within the region had genetic interactions to 10 well-known NMD factor genes, compared to 383 control genes outside the region (Additional File 1: Fig. S22B-C). In this CRISPR analysis, *NOP58* (one of the NMD-related genes, FDR = 7.2%), *BARD1* (9.4%), *SF3B1* (FDR = 16%), *CWC22* (12%), and *FARSB* (16%) were significant at FDR < 25% (one-sided Mann-Whitney *U* test), plus 8 additional genes not known to be NMD-associated (Additional File 1: Fig. S22B), two of them overlapping with our set of 14 candidates (*PRKRA* and *CTDSP1*). Overall, the somatic CNA gains in the 2q31.1-2q36.3 region may also alter the expression of various RNA processing genes, associated with alterations in NMD efficiency.

Text S6: NMD efficiency modulates selection of somatic nonsense mutations

Previous research[11,44] reported that somatic nonsense mutations (producing PTCs) are positively selected specifically in NMD-triggering regions of tumor suppressor genes (TSGs) and they may be negatively selected in oncogenes (OGs). This led us to ask whether the ”global” NMD efficiency of an individual, in addition to the NMD efficiency of specific PTCs in the individual's genome, provides context for modeling the selection upon somatic PTCs. Specifically, we tested whether there is stronger positive selection for NMD-triggering PTCs among individuals/tumors with high NMD efficiency, compared to individuals/tumors with low NMD efficiency, plausible due to the tumors' ability to utilize NMD for ablating the activity of TSGs. Serving as a negative control, the missense mutations would not be anticipated to show a notable difference in positive selection between individuals with higher versus lower iNMDeff. Regarding oncogenes, we do not expect positive selection on somatic PTC except in rare exceptions, while there might be modest negative selection instead[11,163].

To test our hypothesis, we employed the standard dN/dS tool (dNdScv method)[159] that compares observed mutation counts in a gene to an expectation modeled from covariates (e.g. gene expression) and from synonymous mutation counts. Here we focused on nonsense somatic mutations (splitting by NMD-triggering and NMD-evading PTCs) and missense somatic mutations (serving as negative controls since they should not directly trigger NMD),

within the whole TCGA cohort of tumor exomes (see Methods). A dN/dS ratio above 1 for a gene indicates positive selection, while a ratio below 1 signifies negative selection. The analysis involved stratifying patients based on the median pan-cancer iNMDeff into high and low groups and comparing dN/dS, i.e. estimating conditional selection on the cancer genes depending on iNMDeff. Moreover, we studied 50 TSGs and 52 OGs (see Methods). Briefly, we started with the 727 cancer genes from the CGC list[151], and intersected them with the genes with experimental evidence they act as TSGs or OGs ("STOP" and "GO" genes[157]), and with the significant genes (q-value < 0.01) from the Mutpanning set[158]. This led to a core set of 102 higher-confidence cancer genes.

For TSGs, we first checked missense mutations, which normally do not trigger NMD. There was no significant difference in selection between high and low iNMDeff individual groups, defined either by the ETG method (Fig. 6A, right panel) or the ASE method (Additional File 1: Fig. S26A, right panel) for iNMDeff (missense mutations located in NMD-triggering regions ETG dN/dS = 1.17 vs 1.20, p = 0.35, and NMD-evading regions dN/dS= 1.02 vs 1.10, p = 0.61, one-sided Wilcoxon paired tests). However, in the case of nonsense (PTCs) mutations, we observed a difference with contrasting directions: genes with NMD-triggering PTC variants exhibited a significant trend towards higher positive selection in tumor samples with high ETG iNMDeff, whereas tumors with low ETG iNMDeff demonstrated no positive selection (dNdS = 1.64 vs 1.33, p = 4.7e-02, Fig. 6A, left panel), thereby suggesting that individual-level, global NMD efficiency can indeed shape selection on cancer genes (ASE iNMDeff: p = 0.13, Additional File 1: Fig. S26A, left panel).

We also observed a high positive selection in the NMD-evading nonsense mutations for the group of samples with low iNMDeff as well as the group of samples with high iNMDeff, presumably because many of the truncating mutations are deleterious to protein function regardless of NMD silencing at mRNA level. An explanation for the observed trend is that, simply, these truncating mutations lead to complete loss-of-function of the protein even without mRNA being degraded; indeed many truncations would be expected to do that. Some of the truncations might even have dominant-negative effects, where the truncated version of the protein blocks function of the wild-type allele[74,75].

Further, when examining the specific genes driving this higher positive selection in TSGs via NMD-triggering PTCs (Additional File 1: Fig. S26B-C), we identified the top genes with dN/dS differences (> 1) between high and low ETG iNMDeff groups: *SMAD4* (8.4), *TP53* (8.36), *APC* (3.10), *XPA* (2.46), *CDKN1B* (1.52), *FAT1* (1.17), *NRG1* (1.09), and *EP300* (1.06); for these genes, there is evidence that global NMD efficiency boosts positive selection. In contrast, genes with the most negative dN/dS differences (< -1) were *ARID1A* (-1.27), *BCOR* (-1.89), *RB1* (-2.54), *PTEN* (-4.56), and *SMAD2* (-6.14), and in those genes there is no evidence that iNMDeff affects positive selection.

In the case of OGs (Additional File 1: Fig. 26D-E), for nonsense mutations, NMD-triggering PTCs trended towards negative selection overall, but no significant differences were noted between high and low ETG iNMDeff groups (dN/dS = 0.76 vs 0.63, p = 0.42).

In a cancer-type specific analysis, only STAD had significant differences after FDR adjustment (FDR = 1.2%). Nonetheless, trends of higher positive selection on TSG in the ETG high iNMDeff samples (p < 0.25) were seen in other tumors STAD_MSI (p = 7.6e-2), UCEC_POLE

(p = 0.25), lung cancers LUAD (p = 0.19) and LUSC (p = 0.14), GBM (p = 0.28) and COAD_MSI (p = 0.23); many of these cancer types typically have high mutation burdens.

In summary, our data suggests that higher individual-level NMD efficiency enables more effective positive selection of PTCs in TSG, including, prominently, the master tumor suppressor gene *TP53*.
